# Differential regulation of gene co-expression modules in muscles and liver of preterm newborns

**DOI:** 10.3389/fcell.2025.1645959

**Published:** 2025-09-30

**Authors:** Petra Janovska, Tatyana Kobets, Lenka Steiner Mrazova, Michaela Svobodova, Marketa Tesarova, Pavel Kopecky, Petr Zouhar, Martin Rossmeisl, Viktor Stranecky, Stanislav Kmoch, Jan Kopecky

**Affiliations:** ^1^ Laboratory of Adipose Tissue Biology, Institute of Physiology of the Czech Academy of Sciences, Prague, Czechia; ^2^ Metabolomics Service Laboratory, Institute of Physiology of the Czech Academy of Sciences, Prague, Czechia; ^3^ Research Unit for Rare Diseases, Department of Pediatrics and Inherited Metabolic Disorders, 1st Faculty of Medicine, Charles University, Prague, Czechia; ^4^ Laboratory for Study of Mitochondrial Diseases, Department of Pediatrics and Inherited Metabolic Disorders, 1st Faculty of Medicine, Charles University and General University Hospital in Prague, Prague, Czechia; ^5^ Department of Gynaecology, Obstetrics and Neonatology, 1st Faculty of Medicine, Charles University and General University Hospital in Prague, Prague, Czechia

**Keywords:** tissue transcriptome, human, premature newborn, WGCNA, mitochondria

## Abstract

**Background:**

Newborns undergo rapid metabolic and organ adaptations after birth, which are compromised in premature newborns, leading to adverse health outcomes. Molecular mechanisms underlying these transitions remain poorly understood due to limited tissue availability. To address this gap, we characterized tissue transcriptomes using autopsy samples from a unique newborn cohort.

**Methods:**

We analyzed liver (LI), heart (HM), and skeletal muscle (SM) transcriptomes using RNA sequencing in 41 predominantly premature newborns who died shortly after birth. Nearly 14,000 protein-coding gene transcripts per tissue were detected.

**Results:**

Tissues exhibited distinct expression profiles, with LI showed the highest number of tissue-specific genes. SM gene expression correlated strongly with gestational age at birth (i.e., the prenatal development), while LI was influenced by the duration of postnatal survival (i.e., the postnatal development). HM displayed minimal changes, suggesting stable myocardial metabolism during the perinatal transition. Weighted Gene Co-expression Network Analysis (WGCNA) identified tissue-specific gene co-expression modules linked to clinical traits such as gestational age, birth weight, survival duration, nutrition, and exposure to catecholamine treatment. The key functional annotations, validated by differential expression analysis, revealed that LI and SM modules were enriched for mitochondrial metabolism and oxidative phosphorylation genes, with more pronounced prenatal development in SM, and a postnatal increase in both tissues. Data suggests that energy metabolism in SM matures first, followed by the development of muscle functions. Hepatic modules were associated with a postnatal increase in the steroid hormone/xenobiotic metabolism, and a decline in hematopoietic activity. Robust annotations to ribosome activity suggested tissue-specific changes in protein synthesis, which declined prenatally in SM, postnatally in HM. Notably, the supply of exogenous glucose and nutrition type were strongly associated with hepatic gene expression, highlighting the central role of the liver in postnatal metabolic adaptation.

**Conclusion:**

Overall, our study highlights tissue-specific perinatal gene regulation, with mitochondrial maturation emerging as a crucial driver of postnatal adaptation, explaining vulnerabilities in preterm infants. We provide a unique resource for characterizing developmental changes in tissue transcriptomes during the fetal-to-neonatal transition in human newborns.

## Introduction

The perinatal period in humans represents a very dynamic part of ontogenesis. Like other altricial species, humans are born relatively immature compared to precocial placental neonates. Maturation of most organs and systems in humans occurs postnatally (Ferner et al., 2017). Sufficient organ development during fetal life, interlinked with physiological postnatal adaptation to the extrauterine environment, including, e.g., the ambient temperature (Cannon and Nedergaard, 2004) or nutrition (Koldovsky et al., 1995), is a prerequisite for a newborn’s health (Hillman et al., 2012).

Despite inherent complexity, early postnatal human development commonly proceeds without major complications. However, about 10% of newborns are born prematurely, i.e., before the 37th week of gestation (**GW 37**) (Lawn et al., 2023). The threshold of fetal viability is around GW 22–23. Preterm birth and its complications are the leading causes of death in children under 5 years of age (Perin et al., 2022; Lawn et al., 2023). Newborns delivered before GW 28 (i.e., “extremely preterm newborns”) often have long-term health problems, including developmental, neurological, and metabolic disorders (Lawn et al., 2023; Deprez et al., 2024). The risk of chronic adverse health outcomes increases significantly with lower gestational age at birth (referred to further as **Gestation**). Molecular correlates of early postnatal human development physiology and pathology are poorly characterized due to the limited availability of the appropriate tissue samples.

Quantitative analysis of the whole transcriptome using RNA-sequencing (**RNA-Seq**) provides a reliable tool to characterize the development of organs and tissues. This approach is widely used in animal research, e.g., in characterizing mouse liver development from fetal to adult stages (Li et al., 2009; Renaud et al., 2014). However, data on the ontogeny of the whole transcriptome of human tissues and organs are scarce. Developmental changes of the transcriptome across several organs of six mammalian species and a bird were characterized (Cardoso-Moreira et al., 2019); this study also included humans, with samples collected from fetuses between GW 4–20, infants aged 6–9 months, and older healthy individuals. The ongoing Genotype-Tissue Expression project (**GTEx**) aims to characterize tissue-specific transcriptomes from 54 non-diseased tissue sites across nearly 1,000 adult individuals [Mele et al. (2015); https://www.gtexportal.org/home/]; changes in gene expression in multiple tissues during development will also be studied; however, no results are available yet. Other studies characterized gene expression in the human heart (Iruretagoyena et al., 2014; Pervolaraki et al., 2018; Cui et al., 2019; Cao et al., 2020), liver (Yu et al., 2001; Popescu et al., 2019; Segal et al., 2019; Cao et al., 2020), kidney (Menon et al., 2018; Wang et al., 2018; Hochane et al., 2019; Cao et al., 2020), retina (Lu et al., 2020), brain (Darmanis et al., 2015), and some other tissues (Gao et al., 2018; Cao et al., 2020) collected mostly from abortions during the first half of intrauterine life, and very seldom between GW 20–33 (Gao et al., 2018; Wang et al., 2018; Lu et al., 2020).

To our knowledge, no previous studies have examined the whole human tissues’ transcriptome during the critical first hours and days after birth, when rapid metabolic and organ changes occur. To bridge this gap, we took here advantage of a unique biobank of autopsy tissue samples from mostly premature newborns. All died within 3 months, mostly within several hours or days after birth (Brauner et al., 2001; Brauner et al., 2002; Brauner et al., 2006; Hondares et al., 2014; Janovska et al., 2025). Expression of only selected genes has been analyzed across various tissues using this biobank, documenting the influence of Gestation, length of survival after birth (referred to further as **Survival**), and newborn nutrition on gene expression (Brauner et al., 2001; Brauner et al., 2002; Brauner et al., 2006; Hondares et al., 2014; Janovska et al., 2025). Here, we examined transcriptomes of the liver (**LI** *)*, left ventricle myocardium (heart muscle, **HM**), and skeletal muscle (**SM** *; musculus quadriceps femoris*) of the newborns using RNA-Seq. We aimed to characterize the spectrum and dynamics of protein-coding genes (**PCGs**) expression across the selected tissues, examining their dependence on Gestation, Survival, and other traits. The primary goal of this study was to provide a previously unavailable data resource to further elucidate various aspects of early postnatal human development. Focusing here on complex dataset analysis, we prioritized tissue-specific gene expression differences and their trait interactions within defined gene co-expression modules rather than exploring deep underlying biological mechanisms. However, our recent study on the postnatal decline of hepatic hematopoiesis builds upon this work using the information about selected transcripts (Janovska et al., 2025).

## Materials and methods

### Human tissues

**FIGURE 1:**
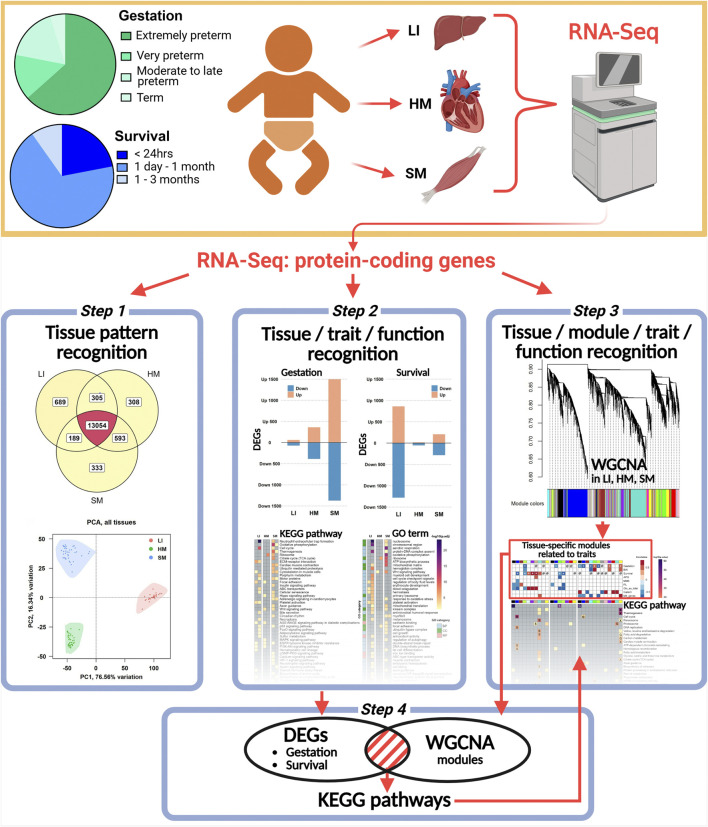
Overview of the experimental and analytical approach used in this study. The schematic depicts sample grouping based on gestational age at birth (Gestation) and length of survival after birth (Survival). RNA-Seq analysis was performed on samples from LI, HM, and SM. Data analysis included: (*Step 1*) tissue-specific gene profiling and PCA; *(Step 2*) differential expression analysis to identify DEGs associated with various traits, followed by functional enrichment analyses using the entire dataset; (*Step 3*) WGCNA to identify key tissue-specific modules related to tested traits and the associated biological pathways and processes in each tissue; and (*Step 4*) validation of DEGs contained within these modules.

The workflow of this study is shown in Figure 1. Autopsy **s**amples of LI, HM, and SM were obtained from human newborns (*n* = 41; mostly premature newborns) within 2–3 h after death. Autopsies were performed obligatorily in these cases. These newborns died during the years 2000–2006 due to various causes, relatively early after delivery at the Division of Neonatology, Department of Obstetrics and Gynecology, General Faculty Hospital and the first Faculty of Medicine, Charles University, Prague, Czech Republic (Table 1; Supplementary Table S1A; Supplementary Figure S1). See also our previous publications based on this biobank (Brauner et al., 2001; Brauner et al., 2002; Brauner et al., 2006; Hondares et al., 2014; Janovska et al., 2025), in which some of the cases used in this study were examined (*n* = 35; Table 1). Tissues from newborns of mothers with endocrinological disorders or drug abuse were not included. The study protocol conforms to the ethical guidelines of the 1975 Declaration of Helsinki, and it was approved *a priori* by the Committees of Medical Ethics at all the collaborating institutions (see the Code of the Ethics Committee of the General University Hospital Prague: 70/18 Grant AZV VES 2019 1. LF UK). Written informed consent was obtained from the parents. Tissue samples were stored in RNA*later* (Ambion, Austin, TX, United States) at −80 °C. Some LI samples are also stored in paraffin blocks for histology (Brauner et al., 2001; Janovska et al., 2025). The biobank is located at the Institute of Physiology of the Czech Academy of Sciences, Prague.

**TABLE 1:** Cases examined.

Case	Sex	Gestational age at birth (weeks + days)	Birth weight (g)	Survival (days)^a^	Multiple birth	Tissue sample for RNA-seq			Clinical and pathological diagnoses
						LI	HM	SM	
Extremely preterm newborns (< GW 28)									
A97^b^	F	20 + 3	350	0		+	+	+	LBNS
A100^b^	M	22 + 0	510	4	G	+	+	+	RDS, ICH
A93^b^	M	22 + 2	500	1.9	G	+	+	+	IA, RDS, ICH
A94^b^	M	22 + 4	450	11.6		+	+		RDS, S, MOF, ICH
A108^b^	M	22 + 4	590	0.6	G	+	+	+	RDS, ICH
A107^b^	F	22 + 5	460	6.7	G	+	+	+	RDS, PDA, NEC, S, ICH
A82^b^	M	23 + 2	690	14.1		+	+	+	RDS, PH, S, M
A113^b^	M	23 + 3	520	0		+	+	+	LBNS, IA, P
A104^b^	F	23 + 4	530	0		+	+	+	LBNS
A96^b^	M	23 + 6	700	1.9		+	+		RDS, ICH
A72^b^	M	24 + 0	680	26.7			+	+	IA, ICH, NEC, MOF
A106^b^	M	24 + 0	620	3.2		+	+	+	IA, RDS, S, ICH
A99^b^	F	24 + 1	320	0		+	+		LBNS
A71^b^	M	24 + 3	680	2			+	+	LH, PPROM, RF, ICH
A101^b^	F	24 + 3	580	6.9	T	+	+	+	RDS, PDA, ICH
A102^b^	F	24 + 3	610	20	T	+	+	+	RDS, PDA, BPD, S, ICH
A77 ^b^	M	24 + 4	690	3.5	G	+	+	+	RDS, ICH
A90^b^	M	24 + 4	985	17.3	G	+	+	+	RDS, PH, S, ICH
A84^b^	F	24 + 6	630	18.8		+	+	+	RDS, ICH
A74^b^	F	25 + 0	380	5.7			+		FGR, PDA, PH, S, ICH
A73^b^	M	25 + 2	435	69.4	G			+	FGR, RDS, ICH
A112^b^	F	25 + 2	700	35		+	+	+	RDS, S, M, ICH
A114^b^	M	25 + 5	750	10.9		+	+	+	RDS, S, NEC
A105	F	26 + 4	830	36.1	G	+	+	+	RDS, PDA, NEC
A92^b^	M	27 + 1	820	29.6		+	+	+	RDS, RF, ICH
A111	M	27 + 2	870	3.7		+	+	+	RDS, S, ICH
Very preterm newborns (GW 28 to < GW 32)									
A87^b^	M	28 + 2	980	2.7		+	+	+	PPROM, S, ICH
A95	M	28 + 4	780	2.6	G	+	+	+	IA, LH, PH, VA
A110^b^	M	28 + 4	1,000	20.6		+	+	+	RDS, PDA, NEC, MOF
A91^b^	M	29 + 5	830	2.4		+	+	+	IA, FGR, ICH
A117	F	30 + 0	1,150	2.6		+	+	+	RDS, ICH
A75^b^	M	30 + 2	1,295	9.6		+	+	+	M, S
Moderate to late preterm newborns (GW 32 to < GW 37)									
A115^b^	M	32 + 0	2060	1.7		+	+	+	RDS, S, ICH
A76^b^	M	32 + 1	750	6.9	G	+	+	+	DS, S
A98^b^	M	32 + 3	1840	0.4	G	+	+	+	LBNS, JS, RF
A70^b^	F	34 + 6	1950	13			+	+	MOF, NEC, ICH, RDS
A85	M	35 + 2	560	**10 min**	G		+	+	LBNS, IA, FGR, ICH
A109^b^	M	35 + 2	1860	0.1		+	+	+	LH, RF
A88^b^	F	36 + 3	1790	89.7	G	+	+	+	FGR, RF
Term newborns (> GW 37)									
A86	F	37 + 5	1,690	13.3		+		+	FGR, LH, NEC
A103^b^	F	38 + 5	1,500	0.8		+	+	+	FGR, LH, PH, RF

Cases in the cohort (*n* = 41) were sorted and classified according to gestational age at birth (Gestation; https://www.who.int/news-room/fact-sheets/detail/preterm-birth).

Abbreviations: F, female; M, male; GW, gestational week; G, gemini; T, trimini; BPD, bronchopulmonary dysplasia; DS, Down syndrome; FGR, fetal growth restriction; IA, intrauterine asphyxia; ICH, intracranial hemorrhage; JS, Jeune syndrome; LBNS, live-born infant not supported after delivery; LH, lung hypoplasia; M, meningitis; MOF, multiorgan failure; NEC, necrotizing enterocolitis; P, pneumonia; PDA, patent ductus arteriosus; PH, pulmonary hemorrhage; PPROM, preterm premature rupture of membranes; RDS, respiratory distress syndrome; RF, respiratory failure; S, sepsis; VA, VATER syndrome.

^a^ In days, except when indicated otherwise (in bold type); “0” marks newborns showing signs of life, in which resuscitation was withheld for various reasons.

^b^ Cases (*n* = 35) examined in our previous studies (Brauner et al., 2002; Brauner et al., 2006; Hondares et al., 2014).

^+^ Samples of LI (*n* = 35), HM (*n* = 39), and SM (*n* = 37) used for RNA-Seq, after the exclusion of degraded RNA isolates (RIN < 6.1). For quality and codes of the RNA isolates, and for various clinical traits, see Supplementary Table S1; for the diagnoses frequency, see Supplementary Figure S1D.

### RNA isolation and quality control

Total tissue RNA was isolated using the RNeasy Mini Kit (Qiagen, Valencia, CA, United States). Typically, 30–100 µg total RNA was obtained from 40 to 50 mg of the tissue. Its quality was checked using 250 ng RNA and the Agilent 2100 Bioanalyzer (Agilent Technologies, Inc., Santa Clara, CA, United States) to obtain the RNA Integrity Number (**RIN**) as the marker. Only samples with RIN > 6.1 were used for RNA-Seq.

### RNA-seq analysis

Complementary DNA library preparation KAPA RNA HyperPrep Kit with RiboErase KK8560 (F. Hoffmann-La Roche, Basel, Switzerland) was used for RNA-Seq library preparation with 1 µg RNA per sample. Sequencing was performed using NovaSeq6000 (Illumina, San Diego, CA, United States). The resulting FASTQ files were subjected to QC control and trimmed using Atropos (version 1.128). An average of 28.38 million 2 × 100 bp paired-end reads were generated per sample, and more than 97% of these reads were mapped to the human reference genome HG38 using STAR aligner (version 2.7.8a). In total, 56% exonic, 36% intronic, and 8% intergenic regions were identified. Gene-level abundances were estimated using Salmon (version 1.3) with the Ensembl gene definition version 94. This resulted in the detection of transcripts corresponding to 51,625 unique Ensembl gene IDs, which included 19,938 only PCGs and 7,629 long noncoding RNAs. In order to reduce the number of false-positive results, transcripts with the sum of raw read counts under 500, calculated across all samples for each gene, were excluded. Thus, only a limited number of transcripts for PCGs were considered for the subsequent analyses, 15,471 PCGs in total, i.e., 14,237 PCGs in LI, 14,260 PCGs in HM, and 14,169 PCGs in SM; long noncoding RNAs were not included. Levels of *UCP3* and *SLC2A4* transcripts measured using qPCR previously (Brauner et al., 2006), similarly as the analysis of *PDK4*, *LIPE* and *FASN* levels in this project, correlated with the values obtained here using RNA-seq (Supplementary Figure S2). The RNA-Seq data were deposited in the ZENODO database under accession number 14045261 [https://doi.org/10.5281/zenodo.14045261; (Stranecky and Kopecky, 2024)].

All downstream calculations were performed in the R environment, version 4.3.0. The PCAtools R package (https://github.com/kevinblighe/PCAtools) was used for PCA on the top 500 variable genes selected from rlog-transformed data. Normalization, rlog transformation, and differential expression (**DE**) analyses were performed within the **DESeq2** R package (Love et al., 2014). This procedure included the median of ratios method, when read counts were divided by sample-specific size factors. Normalized counts were used for DE analysis with the Wald test and Benjamini–Hochberg *p*-value adjustment. Genes with adjusted *p*-value < 0.05 were considered significant; no log_2_ fold change (**log**_**2**_ **FC**) cut-off was applied in case of continuous independent variables, while absolute value of log_2_FC > 0.5 was applied as cut-off after tests with factorized varibales. As the main parameters of interest, Gestation, Survival, and sex were used as independent variables for separate statistical tests. Additional DE analyzes included other traits, specifically, BW, APG, MM3, PL, Glc_su_total, Catech, and Mit_genes (see Abbreviations). For Gestation, Survival, BW, APG, Glc_Su_total and Mit_genes, treated as continuous variables, a standard DESeq2 design formula was specified. DESeq2 fits a generalized linear model for each gene, estimating the association between gene expression and the continuous variable; the Wald test is applied to the coefficient of the continuous variable, and the reported log_2_ fold change corresponds to change in expression per unit increase. To determine genes with preferential expression in one of the tissues, **NOISeq** analysis (Tarazona et al., 2015) was applied to DESeq2-normalized data. The type of replicates was set as “biological.” Expression of genes in one tissue was compared with expression in the other two tissues. Genes with *q*-value > 0.95 and absolute value of log_2_FC > 2 were considered tissue-specific.

### Mitochondrial transcripts abundance

Total transcriptional output originating from mitochondrial genes was expressed as a percentage of sequencing reads aligned to the mitochondrial genome relative to all reads mapped [**Mit_genes**; Garcia et al. (2017); Supplementary Table S1B].

### Gene co-expression analysis and interpretation of RNA-seq data

Analysis of gene expression profiles was performed using Weighted Gene Co-expression Network Analysis [**WGCNA**; ref (Langfelder and Horvath, 2008; Zhao et al., 2010)] based on clustering and correlation approaches. A total bulk of the expressed PCGs in each tissue was segregated into co-expression modules. We performed a “signed” type of co-expression network construction that considered the sign of the correlation information for each pair of individual gene profiles. Thus, each module consisted of clustered PCGs with similar dynamics across tissue. Within each tissue analysis, the procedure assigns each PCG to only one module.

Next, eigengenes were calculated, representing a weighted average of the gene expressions in each module (Langfelder and Horvath, 2008; Zhao et al., 2010), and their profiles were tested for correlation with all recorded traits, such as Gestation, Survival and other clinical parameters, and mitochondrial transcript abundance, using Spearman’s method. Correlation was considered significant at *r*-value > 0.5 or < − 0.5, and *p*-value < 0.05. Co-expression modules with eigengene profiles, positively or negatively correlating with the tested parameters (referred to further as **Selected modules**), were filtered for further annotation. The same approach was used to analyze correlations between tested traits and between eigengenes. A multifactorial linear regression-based approach (Haghani et al., 2023) was also applied, including (i) module eigengene as a dependent variable, (ii) either Gestation or Survival as an independent variable, and (iii) sex as a random factor. In addition, we tested the interaction between Gestation and Survival of the neonate using a separate model that included both these parameters.

Annotation of groups of PCGs from co-expression modules was performed using Over-representation analysis (**ORA**) from clusterProfiler R package (Wu et al., 2021). Information about pathways and terms was extracted from the KEGG (https://www.genome.jp/kegg/) and Gene Ontology (**GO;** https://geneontology.org/) databases.

## Results

### Large heterogeneity of cases examined

The studied cohort was composed of 41 born-alive infants of both sexes (15 females and 26 males), who all died, primarily due to severe prematurity and related pathologies during 2000–2006. These newborns differed concerning Gestation (GW 20.4–38.7) and corresponding birth weight (320–2060 g; Table 1; Supplementary Table S1A). Regarding the international standards (Villar et al., 2014), about 25% of the newborns were “small for gestational age” (Supplementary Figure S1A), which is a higher incidence compared with surviving preterm newborns (5). Most of the newborns (*n* = 26; 63%) were “extremely preterm newborns” (< GW 28), while only two of them (∼5%) were born at term (≥ GW 37; Table 1; Supplementary Figure S1B). The deaths occurred within 24 h (*n* = 9; 22%), mainly between 1 day and 1 month (*n* = 28; 68%), or between 1 and 3 months (*n* = 4; 10%) after birth (Table 1; Supplementary Figure S1C).

The cases were very heterogeneous regarding clinical and pathological diagnoses (Table 1). Frequency of various pathological conditions and their combined presence increased with lower Gestation (Supplementary Figure S1D). Thus, both intracranial hemorrhage and respiratory distress syndrome were diagnosed in 85% of the extremely preterm newborns, with 58% of these newborns exhibiting a combination of these two pathologies. Also, the frequency of sepsis increased with prematurity. On the other hand, the number of newborns with fetal growth restriction and lung hypoplasia correlated positively with Gestation (Lawn et al., 2023). All the above data document the extremely pronounced heterogeneity of the studied newborns, inherent with the nature of this unique clinical cohort.

### RNA-seq of LI, HM, and SM identified 15,471 largely overlapping transcripts: tissue-specific expression patterns

Total RNA isolated from autopsy tissue samples was used for RNA-Seq to characterize LI, HM, and SM transcriptomes (Figure 1). Despite the prolonged storage of the samples, the quality of 94% of RNA isolates (Supplementary Table S1B) was sufficient for the analysis (Table 1). In each tissue, close to 14,000 transcripts for PCGs were considered (see Materials and methods).

**FIGURE 2:**
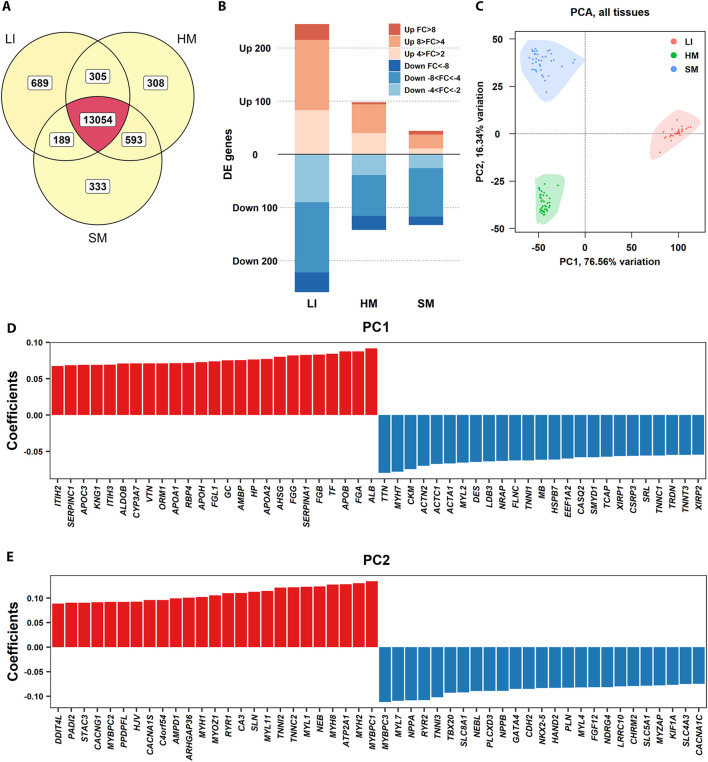
Global patterns of tissue transcriptomes. **(A)** Venn diagram showing the total number of PCG transcripts considered based on RNA-Seq of LI (*n* = 35), SM (*n* = 37), and HM (*n* = 39) samples (Table 1), and their overlap across tissues. **(B)** Bar plot displaying the number of genes with tissue-preferential expression, as determined by NOISeq analysis. The expression level in a given tissue was compared with its combined expression level in the other two tissues. FC refers to log_2_FC (Supplementary Table S2). **(C)** PCA of gene expression profiles, showing the separation of LI, SM, and HM samples based on the top 500 variable genes in three tissues. The first two principal components (PC1 and PC2), explaining the largest proportion of variance, are plotted. **(D, E)** Bar plots showing the genes with the most extreme positive (red) and negative (blue) loadings, indicating genes with the largest contributions to sample separation along PC1 **(D)** and PC2 **(E)**, respectively.

First, we focused on the characterization of the general pattern of gene expression across tissues (Figure 1 – Step 1). About 84% of all these PCGs were expressed in all three tissues, while hundreds of PCGs were expressed exclusively in a given tissue, with a relatively large number in LI, and 15,471 in total considered (Figure 2A). Linear regression analysis of variation within gene expression data indicated a higher contribution of the tissue type factor than the individual (44% vs. 10% of variance explained). NOISeq analysis identified genes non-exclusive to any tissue but with predominant expression in one (Figure 2B; Supplementary Table S2). The number of these genes was approximately twice as high in LI as in HM, with the lowest number observed in SM. Overall, the number of such genes found in newborns in this study was higher than in adult humans (Mele et al., 2015).

The variation among the tissues was confirmed using Principal component analysis (**PCA**; Figure 2C). The principal component 1 (**PC1**, representing 77% of variability) separated the tissues depending on the germ layer of their origin, i.e., (i) LI originated from both endoderm and mesoderm; and (ii) HM and SM, which developed from mesoderm. Separation by PC2 reflected more subtle differences in tissue transcriptomes, probably reflecting muscle fiber structure and metabolism (Blaauw et al., 2013; Lindskog et al., 2015). Plots of top and bottom loadings extracted from PCA results indicated the most discriminating transcripts between LI and muscle tissues (PC1) (Figure 2D), and between HM and SM (PC2) (Figure 2E), respectively. PCGs contributing the most to PC1 encoding hepatic proteins, involved in blood coagulation (*SERPINC1*, *KNG1*, *FGB*, *FGA*), plasma transport (*TF*, *ALB*), lipid metabolism (*APOC3*, *APOA1*, *APOB*), detoxification (*CYP3A7*), acute-phase response (*ORM1*, *HP*); and muscle proteins, crucial for its contraction (*TTN*, *MYH7*, *ACTC1*, *ACTA1*, *MYL2*, *TNN1*, *TNNC1*), structure (*DES*, *LDB3*, *NRAP*, *FLNC*), energy metabolism (*CKM*, *MB*), and cytoskeleton organization (*ACTN2*, *HSPB7*, *CSRP3*, *XIRP1*, *XIRP2*). The major contributors to the PC2 involved PCGs specific for skeletal muscle proteins, such as *MYBPC1*, *MYH2*, and *SERCA1* (*ATP2A1*); and myocardium proteins, such as *MYBPC3*, *MYL7*, and *NPPA*.

The analyses above indicated a relatively high variation in the expression of PCGs among LI, HM, and SM of the newborns, with a relatively high spectrum of LI-specific genes and a major difference between the hepatic and muscle transcriptomes. These data align with the developmental origins and functions of these tissues.

### Interactions between tissue transcriptomes and major traits: SM transcriptome as the most affected

**FIGURE 3:**
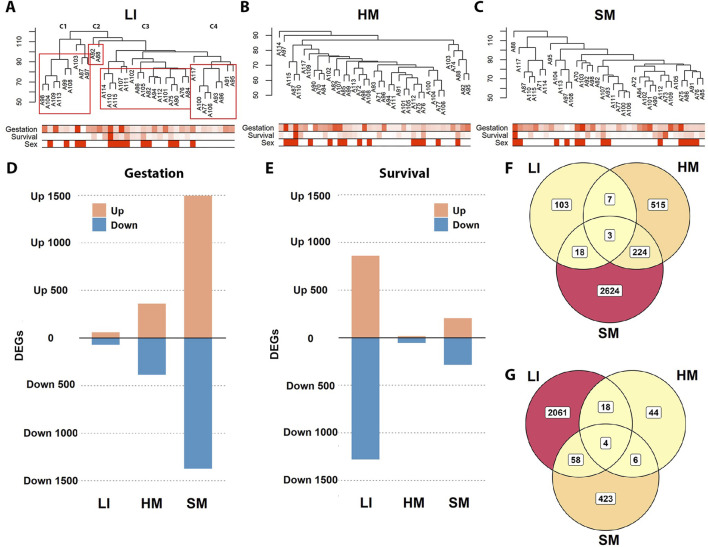
Differential effects of Gestation and Survival on transcriptomes across three tissues. **(A–C)** Two-dimensional hierarchical clustering analyses were performed separately for LI, HM, and SM (dendograms, top). Below each dendrogram is a heatmap representing selected traits: Gestation and Survival are indicated by color intensity (darker shades correspond to higher values), while Sex is denoted by filled cells (males) for each individual sample. In LI, clusters (C1–C4) show a clear association with Survival: C1, median of 0.4 days; C2, >29 days; C3, median of 14.1 days; and C4, median of 3.2 days (see Table 1). **(D, E)** DEGs were identified in each tissue using DESeq2, with Gestation and Survival used as continuous independent variables; for a list of genes, see Supplementary Table S3. **(F, G)** Venn diagrams illustrate the overlap of the DEGs associated with Gestation **(F)** and Survival **(G)** among the three tissues (using data from **(D, E)**).

To assess the effects of the recorded traits on whole tissue transcriptomes and related functions (Figure 1 – Step 2), global two-dimensional hierarchical clustering of gene expression was performed for each tissue separately. In LI, cases are segregated into distinct clusters reflecting Survival. However, Survival had no apparent effect in either HM or SM (Figures 3A–C). In any tissue, the clustering pattern could not be unequivocally explained by Gestation, sex (Figures 3A–C), or any other recorded trait (not shown).

Therefore, next, we sought to characterize directly global changes in tissue transcriptomes with the two major clinical traits, i.e., Gestation and Survival, using DESeq2 analysis (Figures 3D–G; Supplementary Table S3). The number of diferentially expressed genes (**DEGs**) linked to Gestation was the highest in SM, intermediate in HM, and lowest in LI. On the other hand, LI showed the highest number of DEGs linked with Survival, with SM showing less, and HM almost no effect. Considering the effect of both Gestation and Survival together, the SM transcriptome was the most affected, while the HM transcriptome represented the opposite (with 24%, 16%, and 6% of PCGs affected in SM, LI, and HM). DEGs linked to Gestation (Figure 3F) and Survival (Figure 3G), respectively, partially overlapped among the tissues, most prominently the DEGs linked to Gestation between SM and HM, indicating their similar trajectories during prenatal development (Figure 3F).

**FIGURE 4:**
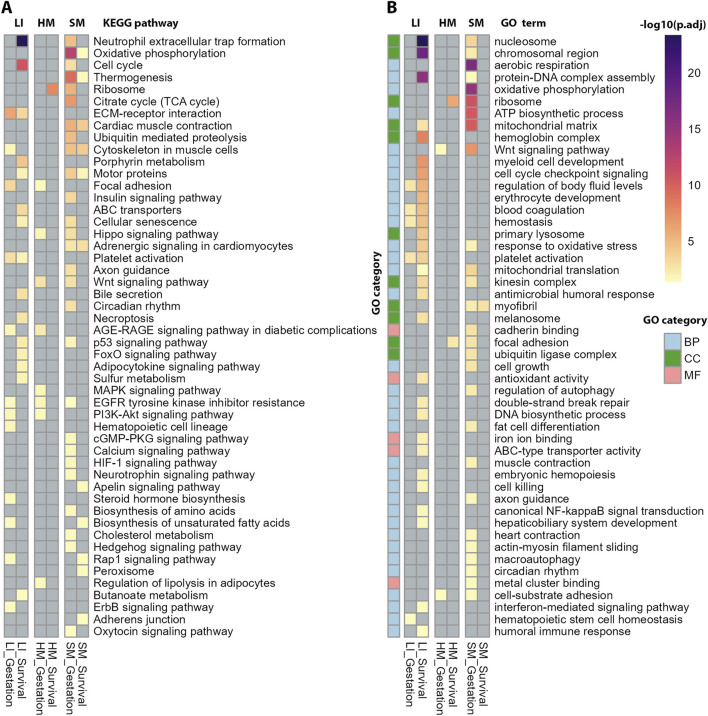
Functional annotation of DEGs associated with Gestation and Survival (corresponding to Figures 3D,E) using ORA. Tissues are shown in columns and annotations in rows. **(A)** The annotation focused on biochemical pathways using KEGG database **(B)** The annotation using GO database across three main categories: Biological Process (BP), Cellular Component (CC), and Molecular Function (MF). The most significant GO terms were selected based on adjusted *p*-values from ORA and their representation across multiple datasets. For complete results of the annotations, see Supplementary Table S4.

Functional annotation of the top DEGs related to Gestation and Survival, respectively, performed using KEGG and GO databases (Figures 4A,B; Supplementary Table S4) suggested tissue-specific association of numerous biological activities with both traits, namely, (i) “mitochondrial activity,” annotated by terms relating to oxidative phosphorylation (**OXPHOS**), mitochondrial metabolism, thermogenesis, and aerobic respiration, and associated cellular processes such as muscle contraction and ribosome activity (KEGG, GO) with Gestation in SM; (ii) cell cycle, ECM-receptor interaction (KEGG), regulation of chromosomal/DNA activity and blood cells formation (GO) with Survival in LI; and (iii) ribosome activity (KEGG, GO) with Survival in HM.

Taken together, the analyses above suggested predominantly (i) prenatal maturation of mitochondrial energy metabolism linked with the muscle contractile system and perhaps other energy-demanding mechanism in SM, and (ii) postnatal changes in cell cycle progression possibly linked with hepatic hematopoiesis in LI. HM transcriptome exhibited only a minimal modulation, limited to the effect of Gestation.

### Tissue-specific sex-biased gene expression linked to infectious diseases of the newborns

**FIGURE 5:**
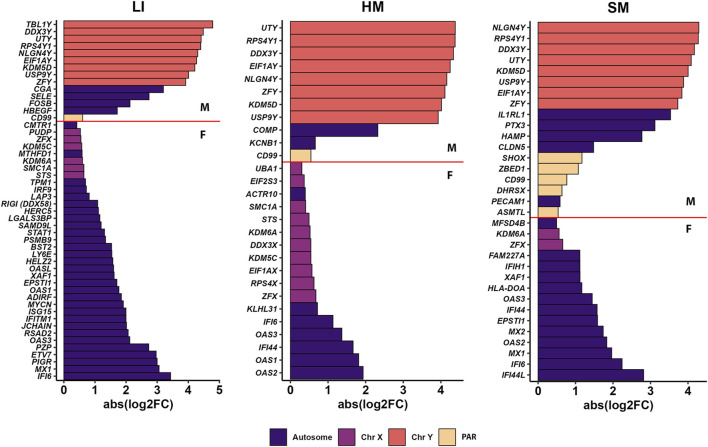
PCGs differentially expressed by sex in LI, HM and SM. For each tissue, bar plots show the top genes with the highest abs (log_2_FC) between mean transcript levels in males (M) and females (F). Genes are color-coded according to their chromosomal location: Autosome, any chromosome that is not a sex chromosome; Chr, chromosome; PAR, genes of the pseudo-autosomal region. For details, see Supplementary Table S5.

We identified 76 PCGs across all three tissues with global sex-biased expression, mostly in LI (50 PCGs) and a lower number in HM and SM (28 and 33 PCGs, respectively; Figure 5; Supplementary Table S5A, B). Of these PCGs, 13 were expressed in each tissue (two on chromosome X, eight on chromosome Y, and one in the pseudo-autosomal region). All the PCGs on sex chromosomes, except for four identified before in adult humans (*EIF2S3*, *SMC1A*, *STS*, *UBA1*) by Melé and colleagues (Mele et al., 2015), are localized in the pseudo-autosomal region or escape X-inactivation and have their paralog on the Y-chromosome. These Y-chromosome paralogs, mostly associated with chromosome remodelation and gene expression machinery, are highly expressed in males in all tissues to compensate for the enzyme dosage of X-linked genes. These genes are usually found as sex-biased in most of the association studies (Zhang et al., 2011; Mele et al., 2015; Cardoso-Moreira et al., 2019).

Functional annotation using the KEGG database of all PCGs with sex-biased expression across all three tissues revealed 12 pathways, with 9 of them related to infectious diseases and 2 to the immune system (Supplementary Table S5C). Next, we performed functional annotation of a subset of 53 PCGs identified exclusively in newborns in this study, and not previously in adults [Mele et al. (2015); Supplementary Table S5D]. Out of 11 annotated KEGG pathways, 10 were associated with infectious diseases or innate immunity, supporting a hypothesis that these associations reflected in part polymorbidity of the newborns studied, namely, the interaction between the effects of sex and infection on gene expression (see Discussion). Indeed, infectious diseases represent a frequent complication and common cause of death in preterm infants (Garvey, 2024). However, very few PCGs with sex-biased expression showed a statistically significant effect of sepsis (not shown).

### Tissue-specific modulation of gene co-expression networks by multiple traits: most of PCGs involved

**FIGURE 6:**
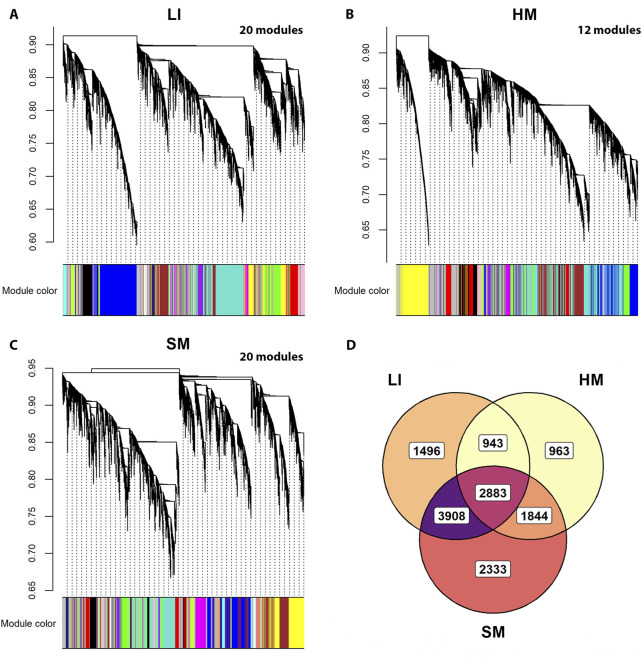
Identification of tissue-specific gene co-expression modules using WGCNA and all RNA-Seq data. **(A–C)** Hierarchical clustering dendrograms representing gene co-expression modules identified in LI, HM, and SM. These modules contained 12,745 genes in LI, 11,760 genes in HM, and 12,764 genes in SM, i.e., 90%, 82%, and 90% of PCGs analyzed per respective tissue. For individual PCGs contained in the modules, see Supplementary Table S6. **(D)** Venn diagram showing the overlap of gene sets among the modules identified in LI, HM, and SM (data from **(A–C)**).

**FIGURE 7:**
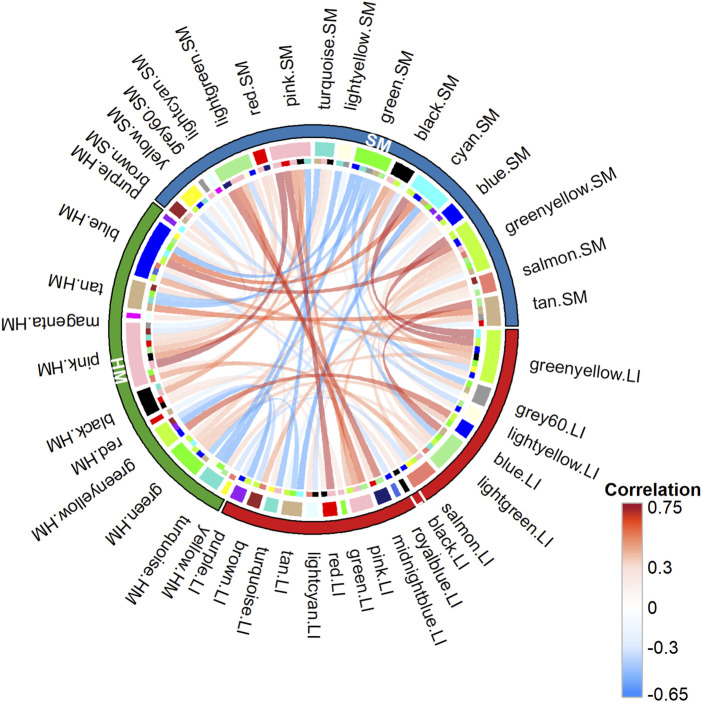
Relationships between WGCNA co-expression modules across tissues. Chord diagram illustrating pairwise Spearman’s correlations between module eigengenes identified in different tissues: 28, LI vs. SM; 19, HM vs. SM; and 13, LI vs. HM. Chords represent significant correlations between eigengenes of different tissue modules (see Supplementary Table S7A).

To recognize trait interactions with tissue transcriptomes in more detail (Figure 1 – Step 3), we used the unsupervised **WGCN**A (Langfelder and Horvath, 2008; Zhao et al., 2010), which clusters most of the expressed PCGs into co-expression modules with similar gene profile. Thus, in each tissue, 12–20 modules were identified (Figures 6A–C). The smallest one contained 63 PCGs (in SM), while the largest module contained 2,693 genes (in HM; Supplementary Table S6). The gene sets composition assigned to the modules partially overlapped among tissues (Figure 6D). The eigengene was calculated for each module as a marker of the expression of all its genes. Inter-tissue correlations between eigengenes of all modules decreased in the following order: (LI vs. SM): > (HM vs. SM) > (LI vs. HM) (Figure 7; Supplementary Table S7A), documenting the relatively strong link between LI and SM metabolism.

**FIGURE 8:**
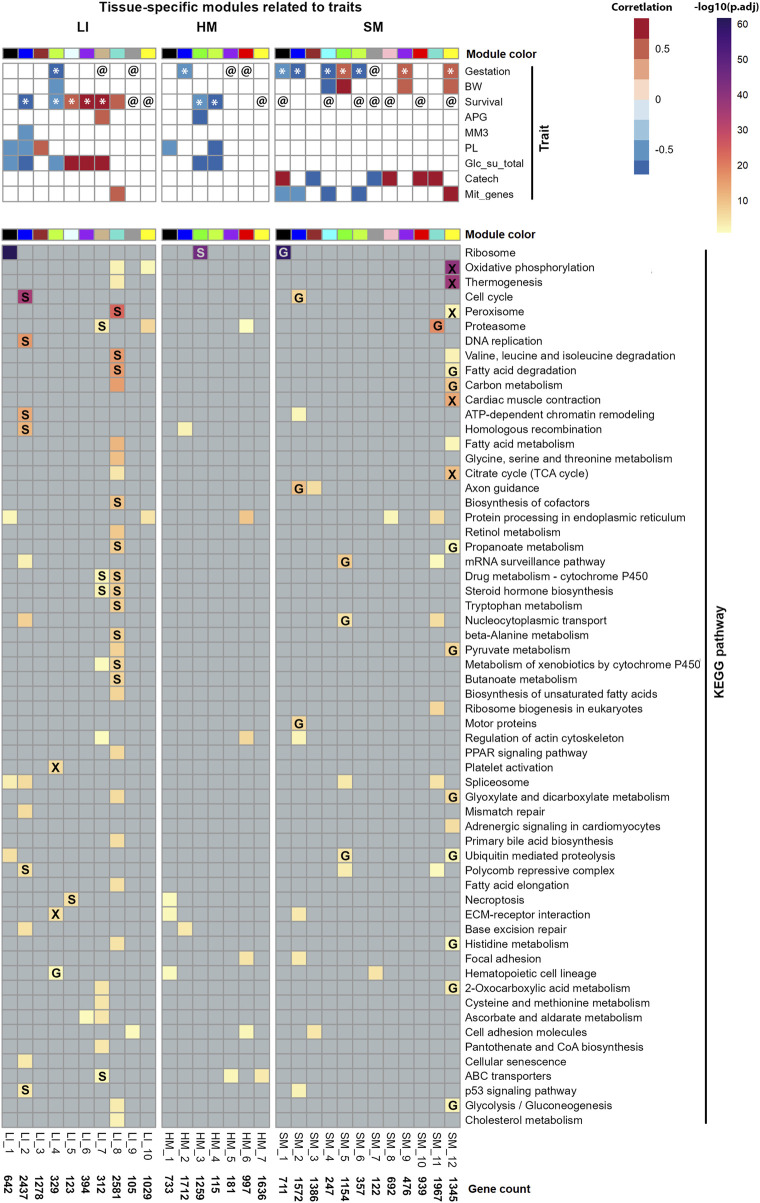
Characterization of Selected modules across tissues. Each column represents a distinct gene co-expression module, each assigned a color according to WGCNA convention. Unique module identifiers and the number of genes per module are indicated at the bottom of each panel (for gene composition of modules, see Supplementary Table S6). Top: Correlations of Selected modules eigengenes and tested traits were assessed using both Spearman’s rank correlation and linear-regression analyses (see Supplementary Table S8). @, module significantly associated with the Gestation or Survival in linear regression (adjusted for sex), but not in correlation analysis; *, modules identified as significant by both approaches. Traits represented in the rows: Gestation, gestational age at birh; BW, birth weight; Survival, survival after birth; APG, Apgar score; MM3 and PL, supplementation with mother’s milk or parenteral nutrition enriched with lipids, respectively, any time during the last 3 days of life; Glc_su_total, the total supply of exogenous glucose during the last 3 days of life; Catech, treatment with catecholamines any time during the last week of life; and Mit_genes, mitochondrial transcript abundance (Supplementary Table S1). Bottom. Functional annotation of each module was performed using ORA and KEGG database (lines); pathways associated with infectious diseases or immunity were not included (Supplementary Table S9; for the corresponding annotation using the GO database; see Supplementary Table S10). Labels in cells: verified effects of Gestation (G), Survival (S), or overlap of both parameters (X; see Supplementary Figure S4; Supplementary Table S11).

Eigengenes of 29 tissue-specific modules correlated with at least one of the recorded traits, i.e., 16 primarily clinical parameters and mitochondrial transcripts abundance (Supplementary Table S1). Conversely, 9 traits related to at least one of the modules (Figure 8). The identified correlating modules (referred to further as **Selected modules**; including those identified additionally using the linear regression approach, see below), i.e., 10 in LI, 7 in HM, and 12 in SM, were assigned unique IDs (the bottom of panels in Figure 8; Supplementary Table S6). These modules contained 65% (LI), 47% (HM), and 77% (SM) of PCGs considered in the respective tissues.

Also in accordance with a relatively low number of PCGs with sex-biased expression (see above; Figure 5; Supplementary Table S5), sex itself did not show a significant correlation with eigengene of any tissue-specific WGCNA-modules. However, a linear regression-based approach that included module eigengene and sex as a random factor, in combination with either Gestation or Survival as a fixed factor (Supplementary Table S8), confirmed the link of several modules to the two tested traits, and revealed additional associations, namely, between Survival and several SM modules (Figure 8).

**FIGURE 9:**
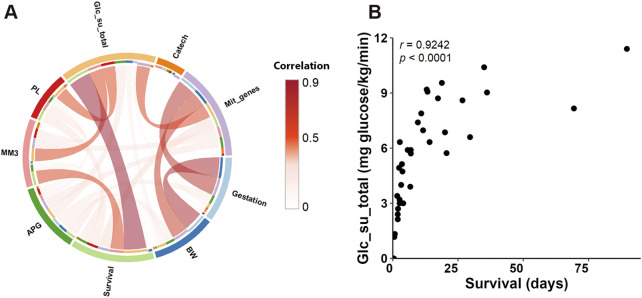
Relationships between traits. **(A)** Chord diagram illustrating pairwise Spearman’s correlations among the traits related to co-expression modules (see Supplementary Table S7B). The width and intensity of the connecting chords indicate the strength of positive correlations between trait pairs. **(B)** Scatter plot showing the relationship between total glucose uptake rate and Survival, with each point representing an individual sample. Spearman’s correlation coefficients (r) and *p*-values are indicated.

Gestation related to a majority of Selected modules in SM, while the number of related modules in both LI and HM was lower (Figure 8). Several SM modules, and one LI module correlated with both Gestation and birth weight. The broader impact of Gestation than birth weight on SM transcriptome is in agreement with the fact that Gestation is more predictive of newborn outcomes than birth weight alone (Del Rio et al., 2020). Most prominently, the major positive effect of Gestation observed in SM, linked also with the effect of birth weight, Survival, and mitochondrial transcripts abundance, was associated with SM_12 module (Figure 8). Thus, this SM gene co-expression module, containing 1,345 PCGs, represents a hub for the above traits, which are mutually correlated (except for Survival; Figure 9A).

Survival mostly correlated with LI modules, unless sex was also considered (Figure 8; see above). Eigengenes of 3 hepatic modules (LI_5, LI_6, and LI_7) correlated positively with both Survival and total supply of exogenous glucose (**Glc_su_total**; for calculation, see Supplementary Table S1), marking the need for glucose supplementation (see Discussion). This trait showed almost unique link with LI transcriptome. This indicated that the correlation between Survival and Glc_su_total, which was the strongest observed correlation between the traits (Spearman’s *r* = 0.92; Figure 9A; Figure 9B; Supplementary Table S7B), reflected in large the hepatic metabolism.

Regarding the newborn nutrition (during the last 3 days of life; see Supplementary Table S1), an exclusive correlation between intake of mother’s milk (**MM3**) and eigengene of a single hepatic “nutritional” LI_2 module, containing a relatively high number of genes (i.e., 2,437 PCGs) was observed, which correlated also with parenteral nutrition enriched with lipids (**PL**), Glc_su_total, and Survival; all these correlations were negative (Figure 8) and mutually correlated (Figure 9A).

Hepatic LI_4 module negatively correlated with a unique set of traits, namely, Gestation, birth weight, Survival, and Glc_su_total, which reflected separate strong positive correlations between (i) Gestation and birth weight (Figure 9A), and (ii) Survival and Glc_su_total (Figures 9A,B; see above). The relatively low number of genes in the LI_4 module (329 PCG) suggests that these genes underlie a relatively small number of functional activities with differential regulation before and after birth (see below).

The routine clinical assessment of neonatal vitality and postnatal adaptation of the newborn using the so-called Apgar score (**APG**) related to only one module in each LI and HM, with both of them related to Survival (Figure 8). The minor effect of APG was consitent with the lack of a correlation between APG and the other traits (Figure 9A; Supplementary Table S7B).

Almost exclusively in SM, a substantial proportion of Selected modules were associated with either (i) catecholamine treatment or (ii) mitochondrial transcripts abundance. The latter reflects both the abundance of mitochondria and the activity of mitochondrial DNA transcription (Garcia et al., 2017). However, the related modules were mostly different (Figure 8). The transcriptome signature of catecholamine treatment, which is frequently used to support the circulatory conditions of the preterm newborns (Ezaki and Tamura, 2012), could reflect the decrease in muscle blood perfusion resulting from dopamine-induced vasoconstriction. On the other hand, that mitochondrial transcripts abundance (Figure 8) correlated with both, Gestation, and birth weight (Figure 9A) agreed with the annotation of DEGs associated with Gestation in SM with OXPHOS and mitochondrial metabolism (Figures 4A,B).

Taken together, the above analysis at the level of gene co-expression modules revealed robust associations between (i) Gestation and SM transcriptome, and (ii) Survival and LI transcriptome, with a less pronounced association between Survival and SM transcriptome. This is consistent with the results DESeq2 analysis of global changes in tissue transcriptomes (Figures 3D–G). Links between most of the other recorded traits and modules across tissues were detected, including the correlation directions between traits and module eigengenes. The results are also highlighting the role of LI in glucose metabolism.

### Functional annotations of genes of selected modules: pronounced tissue-specificity

The comprehensive mapping of trait interactions with tissue transcriptomes at the gene co-expression module level offered deeper insights into the biological significance of the genes involved. To this end, we conducted a functional annotation of the modules genes using ORA, sourcing pathway information from the KEGG (Figure 8) and the GO (Supplementary Figure S3) database (for detailed annotations, see Supplementary Tables S9 and 10).

To minimize potential confounding effects, we excluded gene annotations related to infectious diseases or immune function (Figure 8; Supplementary Figure S3) since infectious disease are of common occurrence in preterm infants [see above, the sex-biased expression; and (Garvey, 2024)]. Despite this conservative filtering approach, a substantial number of annotations were retained. Notably, only a subset of these biological activities could be confidently linked to the specific phenotypic traits associated with each gene module.

Thus, the functional analyses of the gene set contained in the SM_12 module, which positively correlated with Gestation and several other traits (see above; Figure 8), suggested pronounced “mitochondrial activity,” annotated by terms relating to OXPHOS, mitochondrial metabolism, thermogenesis, fatty acid degradation, carbon metabolism, TCA cycle, pyruvate metabolism, *etc.*, as well as peroxisome metabolism, glycolysis/gluconeogenesis, and development of muscle contractile system. The SM_12 module’s robust association with mitochondrial functions, justified its designation as the “mitochondrial” SM_12 module.

On the other hand, prominent negative effects of both Gestation and mitochondrial transcripts abundance, observed in muscle SM_1 and SM_2 modules, were linked with ribosomal (SM_1), cell cycle, axon guidance and other activities (SM_2), suggesting a parallel decrease in overall protein synthesis and muscle cells division prenatally. The association of SM_1 module with both Gestation and Survival suggests its annotated ribosome activity (protein synthesis) may persist to decline postnatally.

The major effect of Survival, observed in LI, could be explained in part by its negative correlations with several activities, which declined postnatally, namely, (i) cell cycle, chromatine remodelation and related activities, including p53 signaling pathway, which were associated with the “nutritional” LI_2 module; and (ii) hematopoiesis and related activities, which were associated with LI_4 module, thus representing a “hematopoietic” module. Both modules correlated with multiple traits (see above; Figure 8).

On the other hand, hepatic activities correlated also positively with Survival. These annotations were namely, represented by (i) interlinked activities involved in metabolism of steroid hormones and xenobiotics [refs. (Faa et al., 2012; Grijalva and Vakili, 2013; Charni-Natan et al., 2019), see Discussion], which were associated with LI_7 and LI_8 modules. While the first module was also annotated with ABC transporters activity (reflecting probably handling of cholesterol, the precursor in synthesis of steroids), the latter module, which was much bigger (312 PCGs vs. 2581 PCGs in LI_7 and LI_8 module, respectively), was linked with more activities, including peroxisomal, mitochondrial, fatty acid and amino acid metabolism; mitochondrial functions were also annotated with LI_10 module. Thus, the dichotomy of the effect of Survival showing both negative and positive correlations with LI gene co-expression modules could be explained by the simultaneous occurrence of different developmental programs in various cell populations in LI during early postnatal development (see Discussion).

The single prominent annotated activity in HM, i.e., the ribosomal activity, was robustly associated with the HM_3 module, which correlated negatively with Survival and two other related traits, suggesting a strong postnatal decline in protein synthesis in HM.

Taken the above results together, the functional annotation at the level of gene co-expression modules revealed groups of genes that underly distinct biological activities, which are differentially linked to tested traits, depending also on the tissue.

### Verification of biological activities linked to gestation and survival in selected modules

Finally (Figure 1 – Step 4), we focused on verifying Gestation- and Survival-related biological activities by intersecting Gestation- and Survival-related DEGs at the whole-tissue transcriptome level (Figures 3D,E; Supplementary Table S3) with gene sets contained in all Selected modules (Figure 8; for the source data, see Supplementary Table S6). Functional annotation of the overlapping genes was perfomed using ORA and KEGG database (Supplementary Table S11; Supplementary Figure S4). In both LI and SM, about a half of the activites annotated to Selected modules were verified, while in HM, only the robust negative effect of Survival on ribosome activity in HM_3 module has been proven (Figure 8).

Some annotations were verified even when the overlapping gene set represented only a small fraction of all module’s genes (Supplementary Table S11). This was observed for the LI_8 module (multiple activities, linked with Survival), and even in the absence of significant trait-module correlations, as for the HM_3 module (ribosomal activity, linked with Survival) and the SM_11 module (proteasome activity, linked with Gestation; Figure 8).

**FIGURE 10:**
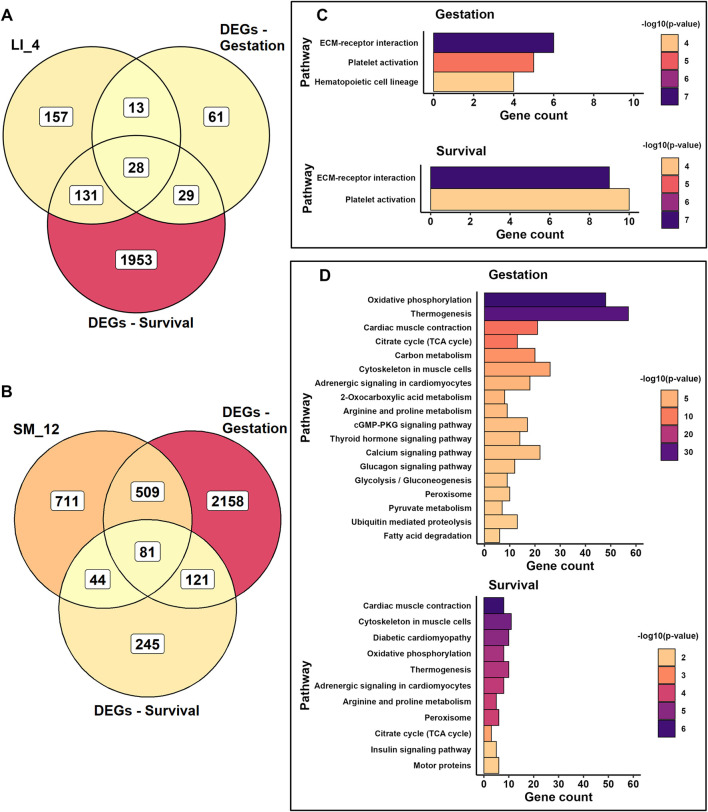
Verification of Gestation- and Survival-associated genes in selected LI and SM modules. **(A, B)** Venn diagrams depict the overlap of gene sets assigned to the LI_4 module **(A)** or SM_12 module **(B)**, and the sets of DEGs associated either with Gestation (DEGs-Gestation) or Survival (DEGs-Survival) as identified in whole tissue transcriptome (see Supplementary Table S6 for module genes; Supplementary Table S3 for DEGs lists). **(C, D)** Bar plots showing KEGG pathways enriched in overlapping genes within the LI_4 module **(C)** and SM_12 **(D)** module, presented separately for the gene overlaps associated with Gestation and Survival, respectively. Gene counts on the x-axis indicate the number of overlapping genes present in each highlighted pathway (see also Supplementary Table S11 and Supplemetary Supplementary Figure S4).

A detailed validation of the activities of two prominent modules is presented in Figures 10A,B. The “hematopoietic” LI_4 module shows substantial overlap with DEGs related to Survival, while the “mitochondrial” SM_12 module overlaps mainly with DEGs associated with Gestation. For the LI_4 module, three activities involved in hematopoiesis were verified. All three activities were affected by Gestation, whereas only two were influenced by Survival (Figure 10C). This occurred despite the fact that the percentage of overlapping genes was higher for Survival than for Gestation in the LI_4 module (Figure 10A). The most significant association with both traits was detected with ECM-receptor interactions, which are integral to hepatic hematopoiesis, particularly during fetal development (Agrawal et al., 2024).

For the SM_12 module, more activities were influenced by Gestation than by Survival (Figure 10D). The strongest associations for both traits were observed with mitochondrial functions, including OXPHOS, thermogenesis, and the TCA cycle, as well as with muscle contraction, cytoskeletal organization in muscle cells, and adrenergic signaling. These findings also indicated that prenatal SM development is primarily linked to the maturation of mitochondrial function, the establishment of glycolysis and gluconeogenesis, and the activation of several signaling pathways. After birth, the main activity in SM is the maturation of its contractile mechanism. However, development of mitochondrial functions after birth was also verified.

## Discussion

Here, we provide a resource of tissue transcriptome data of 41 mostly premature human newborns. All of them died soon, primarily during 28 days after birth. This dataset, obtained using RNA-Seq of LI, HM, and SM autopsy samples, provides information about changes in tissue transcriptomes depending on (i) fetal development during almost the whole second half of the physiological gestational period (i.e., Gestation), (ii) early postnatal development (i.e., Survival), and (iii) several other clinical traits. Robust effects of Gestation and Survival on whole tissue transcriptomes, gene co-expression modules and their annotated activities have been uncovered. To the best of our knowledge, such comprehensive characterization of tissue transcriptomes during early postnatal human development is currently lacking worldwide, primarily due to the scarcity of appropriate biological samples. Our dataset is complementary to the existing resources (Mele et al., 2015; Cardoso-Moreira et al., 2019). Only our dataset is based on the analysis of tissues from born-alive newborns during a relatively narrow time window after birth, which is critical for the postnatal adaptation.

We characterized the development of gene expression in selected tissues during the second major transcriptional change in somatic organs during ontogeny. While the first period occurs during embryonic development and early organogenesis, the second period features increased expression of late organ-specific genes and decreased expression of genes involved in cell division and morphogenesis (Cardoso-Moreira et al., 2019). The variation in PCGs expression among tissues aligned with previous data on adult humans, but individual variability in newborns was relatively high (Mele et al., 2015). This reflects extensive changes in developing organisms compared to adults. The most variable was SM transcriptome, with DEGs linked to both Gestation and Survival. Lower number of DEGs was observed in LI, with almost all of them correlating with Survival. The most stable was HM transcriptome, affected more by Gestation than by Survival. These differences reflected organogenesis, with the heart developing first during fetal life (Tan and Lewandowski, 2020) and maturation of SM proceeding mainly during the second trimester of Gestation, and finishing after birth (Schiaffino et al., 2015). Hepatic hematopoiesis transfers entirely to the bone marrow postnatally, coinciding with the maturation of hepatocyte energy metabolism (Brauner et al., 2001).

Namely, the use WGCNA to detect gene co-expression modules proved useful for the interpretation of the extremely heterogeneous data. Despite the differential effect of Gestation and Survival on LI and SM transcriptome, the correlation between eigengenes of tissue-specific co-expression modules was highest between LI and SM, followed by HM and SM, and lowest between LI and HM (Figure 7). This suggests that LI and SM share more common regulatory mechanisms or metabolic links compared to HM.

The transition to the extrauterine environment following birth provides a substantial physiological stimulus for a whole-body adaptive response, including a switch from energy metabolism based on glycolysis in fetal life to metabolism relying mainly on OXPHOS (Valcarce et al., 1994; Singer and Muhlfeld, 2007; Krizova et al., 2021) fueled increasingly by the mother’s milk lipids. Preterm birth is associated with functional impairment of mitochondria as documented in rat liver by reduced mitochondrial content, OXPHOS activity, and ATP production (Valcarce et al., 1994). In humans, evidence remains largely indirect [reviewed in (Bartho et al., 2020; Mohammadi et al., 2022)].

Transcriptomic analyses in SM performed here identified a key “mitochondrial” module (SM_12) annotated with OXPHOS and other mitochondrial functions, and mitochondrial transcripts abundance, which positively correlated with Gestation, birth weight, and Survival. The “mitochondrial” annotation was verified by the overlap with DEGs linked to both Gestation and Survival, showing the enrichment of these overlaps for OXPHOS and mitochondrial metabolism genes. Moreover, results indicated prenatal maturation of glycolysis/gluconeogenesis and other intermediary metabolism pathways, and their regulatory mechanisms, and that muscle contractile system becomes the major mechanism to be developed postnatally (Figure 10C). These findings demonstrate that during the perinatal period, the energy metabolism matures first, followed by the subsequent development of muscle functions dependent on it, the energy-dependent locomotion (Schiaffino et al., 2015) and thermogenesis (Bardova et al., 2024).

In contrast with SM, LI modules with mitochondrial annotation (LI_8 and LI_10) correlated only with Survival but not with Gestation, and their mitochondrial annotation was relatively weak. However, the postnatal increase of ATP production by mitochondrial OXPHOS is needed for involvement in energy-demanding metabolic activities in the LI, namely, glycogen synthesis, gluconeogenesis, and ketogenesis are essential for the postnatal adaptation of newborns (Hawdon et al., 1992; Singer and Muhlfeld, 2007). These findings underscore tissue-specific mitochondrial maturation patterns, with SM exhibiting stronger developmental ties to gestational age compared to LI, along with an increase in capacity for lipid catabolism and glucose metabolism prenatally (see LI_8 module in Figure 8), ensuring sufficient OXPHOS capacity for postnatal switch from glycolysis to fatty acid oxidation in SM of full-term newborns.

Preterm newborns have labile glucose levels, with a more pronounced decline within the first hours after birth, likely explained by inefficient supply of glucose by both hepatic glycogenolysis and gluconeogenesis in LI (Grijalva and Vakili, 2013). Management of hypoglycemia using glucose infusion (Sharma et al., 2017), as well as parenteral nutrition (Robinson et al., 2023), are the key elements in the early care of preterm newborns. Indeed, our results have shown that the supply of exogenous glucose (Glc_su_total) to maintain euglycemia was primarily linked to the hepatic transcriptome, confirming that insufficient release of glucose from LI was the primary cause of hypoglycemia requiring the glucose supply. The glucose supply and Survival correlated with multiple common hepatic gene co-expression modules, including those linked with mitochondrial metabolism (as noted above), though the primary genetic drivers underlying these relationships remain unresolved. In this context, our results also revealed a strong hepatic transcriptome signature associated with parenteral nutrition and mother’s milk intake.

Postnatal adaptation is controlled in large by steroid hormones, which regulate metabolism, cardiovascular, and respiratory systems. Prenatally, circulating steroid levels are high, and they decrease sharply after birth (Dukic and Ehlert, 2023). This dynamics reflects the neuroprotective role of the steroid hormones (Hirst et al., 2008) and their essential effect in suppressing myometrial contractions (Haoui et al., 2025). Hepatic metabolism of steroid hormones is interlinked with that of xenobiotics through the common use of cytochromes P450 and related enzymes (Faa et al., 2012; Grijalva and Vakili, 2013; Charni-Natan et al., 2019). However, our knowledge about steroid hormones metabolism in LI and its systemic role during the perinatal period is limited (O’Shaughnessy et al., 2013). Two hepatic modules (LI_7 and LI_8), which correlated positively with Survival, verified by the overlap with DEGs, were annotated with steroid hormone/drug metabolism; e.g., the LI_8 module contained *CYP3A4* encoding the most abundant cytochrome P-450 isoenzyme, which is essential for elimination of many drugs and participates in the biosynthesis of steroid hormones (Charni-Natan et al., 2019). Low levels of this protein makes the neonates particulary susceptible to overdosage by many pharmaceuticals (Faa et al., 2012). Moreover, hepatic LI_2 module, which correlated negatively with Survival, was annotated with p53 signalling pathway, which plays a central role in cell fate regulation (i.e., cell-cyle arrest, senescence and differentiation) and also controls hepatic steroidogenesis (Charni-Natan et al., 2019). These data suggested that further characterization of the gene composition of the LI_7, LI_8, and LI_2 modules and the temporal trajectories of these gene expressions will help to understand better the perinatal development of the interlinked hepatic activities engaged in the metabolism of both xenobiotics and steroids. They indicated that hepatic metabolism of steroid hormones might contribute to controlling the circulating level of these hormones. Moreover, the LI_8 module represented the hepatic hub for several activities with verified link to Survival, including peroxisome activity, metabolism of several amino acids, and biosynthesis of cofactors (Figure 8), providing a dataset for the characterization of postpartum development of these activities in LI.

Besides the key role of LI in the above described activities, LI also performs hematopoietic functions. In contrast to the metabolic role of LI, hepatic hematopoiesis is important for fetus and declines postnatally. It is peaking around GW 24–28, before ceasing within the first week after birth as the bone marrow takes over the hematopoiesis (Brauner et al., 2001). In this study, hepatic hematopoiesis was linked with LI_4 module, and possibly also with LI_2 module, which was annotated with cell cycle and related activities; both modules negatively correlated with Survival, while the LI_2 module also correlated with the nutritional components, and LI_4 module also correlated with Gestation/body weight (Figures 8, 10C).

The dynamics of hematopoietic cell lineages during postnatal hepatic hematopoiesis decline remained poorly characterized. To address this, we analyzed cell lineage fate using selected gene expression data from the dataset (Janovska et al., 2025) before presenting the full dataset in this study. We have found coordinated expression of the “erythropoietic marker” *TFRC* and the genes for regulatory and metabolic factors associated with erythropoiesis (*SLC4A1*, *KLF1*, *SLC2A1*, *TAL1*, *GFI1B*, *AHSP*, *UCP2*, *ZFPM1*, *HBA2*, and *HBA1*); all of them with their expression correlated with Survival and mapped to the LI_2 module (Supplementary Table S6), verifying association of this module with hematopoiesis. Expression of the “megakaryopoietic/platelet marker” *ITGB3* correlated with both Gestation and Survival and it was mapped to LI_4 module (Supplementary Table S6), in accordance with the annotation of this module with Platetelet activation (Figure 8). Moreover, expression of the “granulopoietic marker” *MPO* and all transcription factors controlling granulopoiesis tested (*CEBPE*, *CSF3R*, *GFI1*, and *ELANE*) was mapped to yet another LI module (i.e., pink module in all detected modules; Supplementary Table S6). Together with immunohistochemical analysis of the liver of the newborns, characterization of temporal trajectories of the selected genes, based on the dataset described here, revealed that prenatal and early postnatal hepatic hematopoiesis is dominated by erythropoietic cells. These cells are rapidly suppressed within 3 days after birth, contrasting with granulopoiesis’s gradual decline (Janovska et al., 2025). This first detailed characterization of human neonatal hepatic hematopoiesis highlights this resource’s importance and possible uses for characterization of many aspect of human perinatal development.

Robust annotation with ribosome activity revealed pronounced changes in the rate of protein synthesis during perinatal development in each tissue. Negative correlation with Survival in HM (Figure 8) suggests a decline in protein synthesis following what was likely a transient, stress-induced increase during adaptation to extrauterine life, which may not have been detected due to sampling timing. Negative correlation with Gestation in SM suggests a decrease in the activity before birth (Figure 8).

We found here a relatively high number of genes with sex-biased expression compared to adult humans, particularly in LI (Mele et al., 2015). Most of these PCGs were linked to infectious disease or immune pathways, suggesting a possible interaction between sex and infectious disease in affecting gene expression in the newborns. However, such mechanism could not be confirmed because most infants in our cohort had recognized or unrecognized infections, a common cause of death in preterm babies infants (Garvey, 2024). The observed pattern may instead reflect developmental shifts in the immune system gene expression after birth, independent of infection. Evidence from animal studies supports both possibilities (Klein and Flanagan, 2016), while comparable human data - especially at the tissue level - are not available (Winckelmans et al., 2017).

Using WGCNA to analyze highly heterogeneous data allowed us to identify tissue-specific gene co-expression modules linked to the traits studied, assign functional annotations to these modules, and evaluate their biological relevance through module–trait correlations. While this approach is effective for a broad yet detailed overview, it was complemented by analyzing the effects of individual parameters (i.e., Gestation, Survival, and sex–the routinely used clinical characteristics in neonatology; Figures 3, 5) on whole-tissue transcriptomes. Such analyses not only validated module-level functional annotations but also revealed broader interactions between biological activities and traits (see the Results). Therefore, for possible usefulness in understanding the regulation of various functional activities, this complementary approach was performed for all the remaining parameters recorded (i.e., birth weight, APG, MM3, PL, 1017 Glc_su_total, Catech, and Mit_genes) by analyzing their effect on the whole transcriptome of each tissue separately, as documented by Supplementary Figure S5 and Supplementary Tables S12 and 13.

Despite significant advancements in the clinical care of preterm newborns, particularly extremely preterm infants with unique vulnerabilities, further improvements are needed. For example, in Europe, 61% of mortality of newborns (infants under 28 days of age) was attributable to preterm birth in 2020–2021 (Lawn et al., 2023). The gene expression data presented here could aid in identifying targets for better therapies, e.g., if mitochondrial function can be optimized through nutritional or pharmacological interventions, it may enhance the postnatal adaptation of preterm infants by affecting major energy-dependent metabolic pathways involved in both LI and SM. However, any therapy will not be without a risk of adverse side effects. Thus, intervention using catecholamines, used to support the circulation of the preterm newborns (Ezaki and Tamura, 2012), was shown here to broadly interfere with SM transcriptome suggesting large tissue remodeling. Clinical relevance of this effect should be clarified.

Limitations of the study: Various pathological states and the causes of death probably have a significant confounding effect. Although functional annotations related to infectious diseases or immunity were not explicitly included in the data analysis, their effects may still be reflected in other functional annotations - particularly within modules where they were predominantly mapped (i.e., LI_5, HM_3, and SM_3 modules; see Supplementary Tables S5, S9). Interactions between all these factors and the absence of “healthy controls” make interpretation of the data challenging. Transcriptome signatures of various diseased states could not be characterized due to their large number and the relatively small cohort of newborns. Storage of the autopsy tissue samples in RNA*later* (Ambion, Austin, TX, United States) did not allow for the characterization of transcriptome at the single cell level. Furthermore, due to the inherent characteristics of our cohort and the observational design of the study, several important variables could have influenced the tissue transcriptomes. Specifically, a majority of the mothers and/or newborns received pharmacological treatments such as prenatal glucocorticoids, as well as postnatal interventions including glucose administration and antibiotic therapy. Most of the newborns in our cohort were exposed to one or more of these clinical interventions, which may have contributed to variations observed in hepatic and muscle gene expression profiles.

In conclusion, transcriptome analysis of LI, HM, and SM in predominantly preterm newborns, namely, at the gene co-expression module level, revealed major differences between hepatic and muscle gene expression. LI metabolism matures significantly after birth, while SM metabolism develops mainly before birth, with mitochondria playing a crucial role in both tissues for postnatal adaptation, underlying also the compromised adaptation in preterm newborns. The HM transcriptome was the most stable, indicating that myocardium metabolism changes little during the fetal to neonatal transition. This dataset, validated by targeted analysis of hematopoietic gene trajectories, provides a unique valuable resource for characterizing developmental changes in neonatal tissue metabolism and mechanisms operating during the late prenatal and early postnatal period.

## Data Availability

The datasets presented in this study can be found in online repositories. The names of the repository/repositories and accession number(s) can be found below: https://doi.org/10.5281/zenodo.14045261, 14045261.

## Supplementary material

SUPPLEMENTARY TABLE S1 Detailed description of the cohort, clinical traits, identification of tissue RNA isolates and RNA quality, mitochondrial transcript abundance, and verification of transcript quantification using RNA-Seq. (A) Detailed description of the cohort and clinical traits. * Cases (n = 35) examined in our previous studies (Brauner et al., 2003; Brauner et al., 2006; Hondares et al., 2014). F, female; M, male; APG, Apgar score at the10th minute after birth, arbitrary units; MG, multiple gestation (1) or not (0); Delivery, birth via Caesarean section (1) or vaginal birth (0); MM3, supplementation with mother's milk any time during the last 3 days of life (1) or not (0); PNnoF, parenteral nutrition without fat any time during the last 3 days of life (1) or not (0); PL, parenteral nutrition enriched with fat any time during the last 3 days of life (1) or not [0; see Table 1 of ref. (Brauner et al., 2006)]; Catech, treatment with catecholamines (in all cases dopamine containing pharmaceuticals, namely Tenzamine, dopamine; rarely dobutamine and adrenaline) any time during the last week of life (1) or not (0); Corticoids, intervention with hydrocortisone (i.v.), any time during the last week of life (1) or not (0) - either supplementation due to adrenal insufficiency (Subst; 1.5 mg/kg/day), or therapeutical doses (Pharm; >1.5 mg/kg/day); Insulin, treatment with insulin any time during the last 3 days of life (1) or not (0); Glc_load, mean glucose uptake via parenteral nutrition during the last 3 days of life (mg/kg/min); Glc_su_total, mean supply of exogenous glucose during the last 3 days of life (calculated as Glc_load + glucose provided with mother's milk; mg/kg/min). (B) Quality of RNA isolated from LI, HM, and SM of the individual cases, corresponding RNA- Seq data codes, and mitochondrial transcript abundance. LI RNA-Seq, HM RNA-Seq, and SM RNA-Seq, codes of RNA isolates and related RNA-Seq data/libraries (ZENODO database; accession number 14045261); RIN_LI, RIN_HM, and RIN_SM, RIN, RNA integrity number marking quality of the individual RNA isolates used for RNA-Seq (with 1 being the most degraded profile and 10 being the most intact one; LI: 6.2 − 8.8; SM: 7 − 8.9, HM: 6.1 − 9.3). LI Mit_genes, HM Mit_genes, and SM Mit_genes, mitochondrial transcript abundance with average contribution to global transcription in LI: 3.5 ± 0.9; HM: 3.8 ± 1.0; SM: 2.3 ± 0.9%. This number was several-fold lower compared with previous studies in adult humans (Mercer et al., 2011; Mele et al., 2015), reflecting a methodological difference.

SUPPLEMENTARY TABLE S2 Genes with tissue-preferential expression detected using NOISeq.

SUPPLEMENTARY TABLE S3 DEGs associated with either Gestation or Survival identified using DESeq2. Values of log2FC reflected the rate of change in gene expression per unit of independent variable. Assignment of the individual genes to modules identified using WGCNA (see Supplementary Table S6) is also indicated.

SUPPLEMENTARY TABLE S4 Functional annotation of results of DE analysis using ORA, and KEGG and GO database. Functional annotation of the top DEGs associated with either Gestation or Survival (see Supplementary Table S3).

SUPPLEMENTARY TABLE S5 Genes with sex-biased expression in LI, HM, and SM and their functional annotation. (A) List of all genes with sex-biased expression found in each tissue and across all tissues. (B) Summary of results in A; M, male; F, female; Fold_change, fold change between mean transcript level in males vs. females. (C) Functional annotation of all 76 genes with sex-biased expression using ORA and KEGG database. (D) Functional annotation using ORA and KEGG database of all 56 genes with sex-biased expression found here in newborns and not by Mele et al. in adults (Mele et al., 2015).

SUPPLEMENTARY TABLE S6 Genes of all tissue-specific co-expression WGCNA-modules and Selected modules. Modules identified in LI, HM, and SM were assigned different colours according to WGCNA convention. Selected module, tissue-specific WGCNA co-expression module, with eigengene correlating with at least one of the recorded clinical traits or mitochondrial transcript abundance (Supplementary Table S1), or which were identified using a linear regression approach (see legend to Figure 8 Upper panels; Supplementary Table S8); these modules were assigned unique IDs (Module_name).

SUPPLEMENTARY TABLE S7 Correlations between eigengenes of all gene co-expression modules (A) and between tested traits (B).

SUPPLEMENTARY TABLE S8 Linear model-based analysis of the association between module eigengenes and traits with sex as a random factor. Module eigengene and sex were used as a random factor in combination with either Gestation, or Survival, or their combination.

SUPPLEMENTARY TABLE S9 Functional annotation of genes comprising Selected modules in LI, HM, and SM using ORA and KEGG database.

SUPPLEMENTARY TABLE S10 Functional annotation of genes comprising Selected modules in LI, HM, and SM using ORA and GO database.

SUPPLEMENTARY TABLE S11 Functional annotations of genes in Selected modules with verified effect of Gestation or Survival. Genes overlapping between Gestation/Survival-associated DEGs (Supplementary Table S3) and genes in Selected modules (Supplementary Table S6), were analyzed using ORA and KEGG database to identify pathways enriched in both datasets.

SUPPLEMENTARY TABLE S12 DEGs associated birth weight, APG, MM3, PL, Glc_su_total, Catech, and Mit_genes identified using DESeq2.Values of log2FC reflected the rate of change in gene expression per unit of independent variable. For abbreviations, see Figure 8.

SUPPLEMENTARY TABLE S13 Functional annotation of results of DE analysis using ORA and KEGG database. Functional annotation of the top DEGs associated with various traits (see Supplementary Table S12).

## Abbreviations

DE, differential expression; DEGs, diferentally expressed genes; DESeq2, software package in bioinformatics for analyzing RNA sequencing data to identify differentially expressed genes; Gestation, gestational age at birth; Glc_su_total, total exogenous glucose supply during the last 3 days of life (for calculation, see Supplementary Table S1); GO, Gene Ontology; GW, gestational week; HM, left ventricle myocardium; LI, liver; log_2_FC, log_2_ fold change; MM3, mother’s milk any time during the last 3 days of life; Mit_genes, mitochondrial transcript abundance; NOISeq analysis, robust differential expression analysis of RNA-seq data inclucing control for false discovery rates, used here to depict genes with preferential expression in a given tissue; ORA, Over-representation analysis; OXPHOS, oxidative phosphorylation; PCA, Principal component analysis; PL, parenteral nutrition enriched with lipids any time during the last 3 days of life; RIN, RNA Integrity Number; RNA-Seq, RNA sequencing; Selected module, gene co-expression module identified using WGCNA, with eigenegene correlating with at least one of the tested traits, or the trait-module correlation proved using a linear regression approach; SM, skeletal muscle; Survival, length of survival after birth; WGCNA, Weighted Gene Co-expression Network Analysis.
