# Supplementary material for: Differential regulation of gene co-expression modules in muscles and liver of preterm newborns
Source: Front Cell Dev Biol. 2025 Sep 30;13:1645959. doi: 10.3389/fcell.2025.1645959 (PMC12518348; doi:10.3389/fcell.2025.1645959)
Supplement: Supplementary file 5 [file DataSheet1.pdf]

Differential regulation of gene co-expression modules in muscles and liver of preterm newborns

Petra Janovska[1†], Tatyana Kobets[2†], Lenka Steiner Mrazova[1,3], Michaela Svobodova[1], Marketa Tesarova[4], Pavel Kopecky[5], Petr Zouhar[1], Martin Rossmeisl[1], Viktor Stranecky[3], Stanislav Kmoch[3], Jan Kopecky[1*]

Supplementary Figures

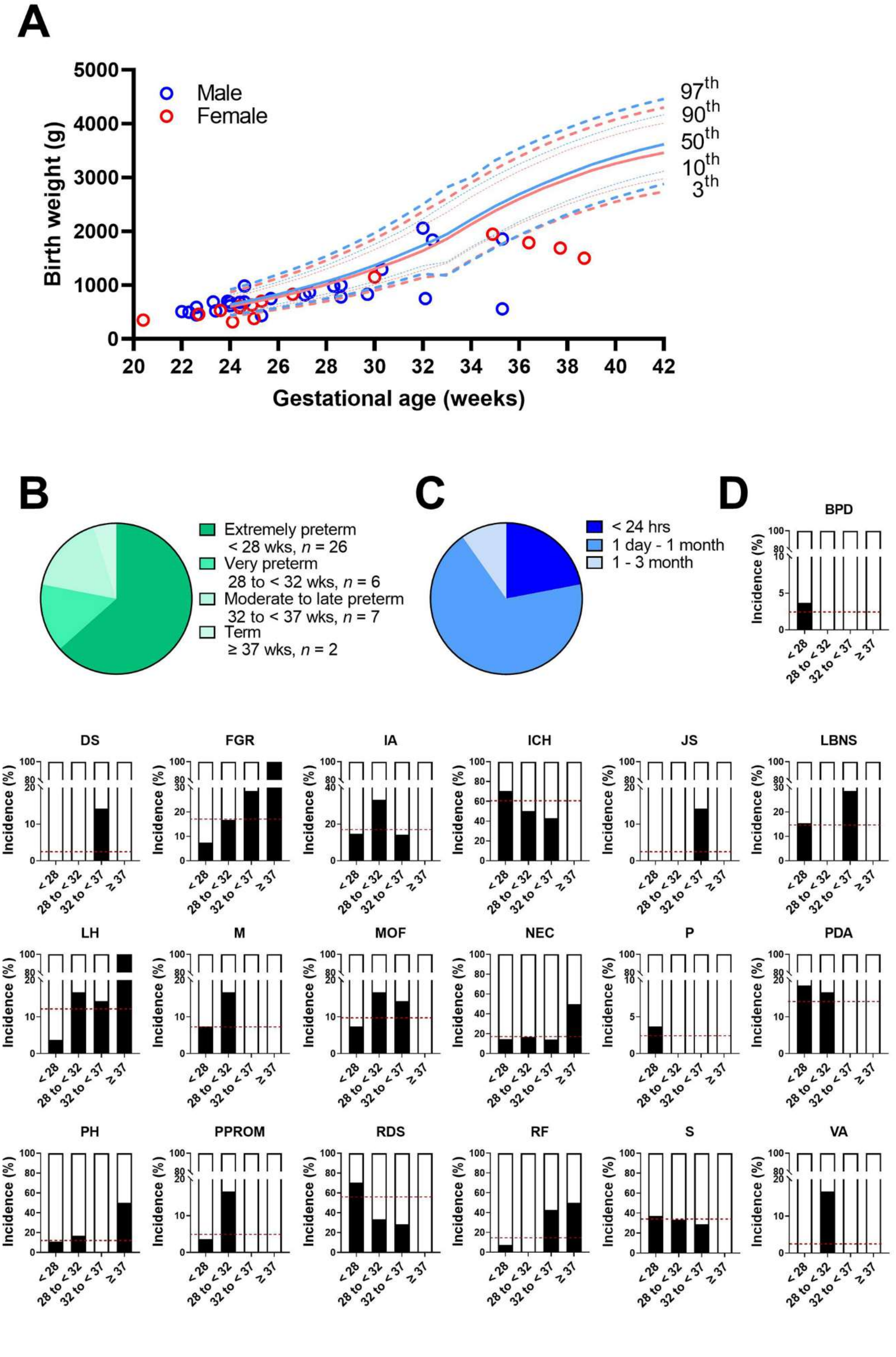
A
Birth weight (g)
Gestational age (weeks)
Male
Female
97th
90th
50th
10th
3th
B
Extremely preterm
< 28 wks, n = 26
Very preterm
28 to < 32 wks, n = 6
Moderate to late preterm
32 to < 37 wks, n = 7
Term
≥ 37 wks, n = 2
C
< 24 hrs
1 day - 1 month
1 - 3 month
D
BPD
DS
FGR
IA
ICH
JS
LBNS
LH
M
MOF
NEC
P
PDA
PH
PPROM
RDS
RF
S
VA
Incidence (%)
< 28
28 to < 32
32 to < 37
≥ 37

S1 Figure *Characteristics of newborn cohort and associated clinical parameters.*
(**A**) Relationship between Gestation and birth weight for all 41 cases examined, representing infants who were born alive but died within three months of age. Standard reference centile curves (3rd, 10th, 50th, 90th, 97th) from a population of low-risk newborns (who mostly survived), according to (Villar et al., 2014), are shown for comparison; infants falling below the 10th centile are classified as small for gestational age. (**B - D**) Distribution of key traits among the 41 cases (% of total): (**B**) Classification of prematurity by gestational age at birth using WHO categorization criteria (see https://www.who.int/news-room/fact-sheets/detail/preterm-birth). (C) Length of survival after the delivery. (**D**) Incidence rates of selected clinical and pathological diagnoses are displayed as percentages within each gestational age subgroup, as described in panel **B.** Each plot shows the proportion of cases with (black bars) or without (empty bars) the diagnosis; the red dashed line indicates the percentage of all 41 cases with the respective diagnosis. For abbreviations, see the legend in Table 1. For further details, see Table 1 and Supplementary Table S1.

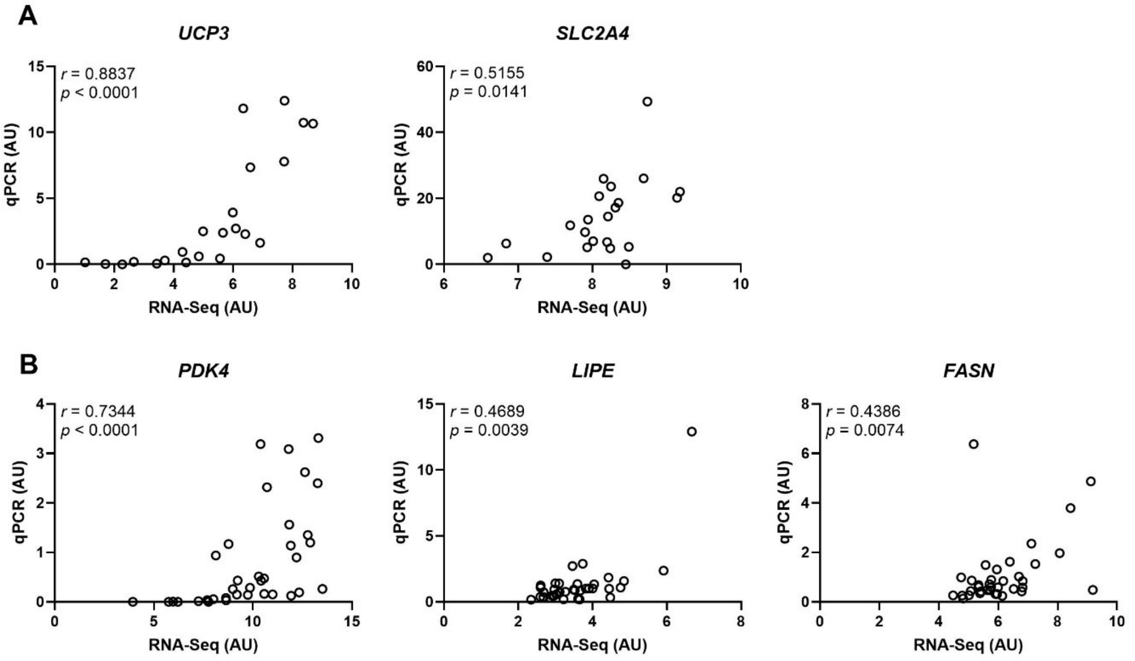

S2 Figure *Verification of transcript quantification by RNA-seq and qPCR.*
(A) Scatter plots depict the correlation between levels of *UCP3* and *SLC2A4* transcripts measured using qPCR as previously reported (Brauner et al., 2006) and those obtained in the present study using RNA-seq. (B) Correlation between levels of *PDK4*, *LIPE,* and *FASN* transcript evaluated using the two methods within this study. Each point represents an individual sample. Spearman's correlation coefficients (r) and *p*-values are indicated.

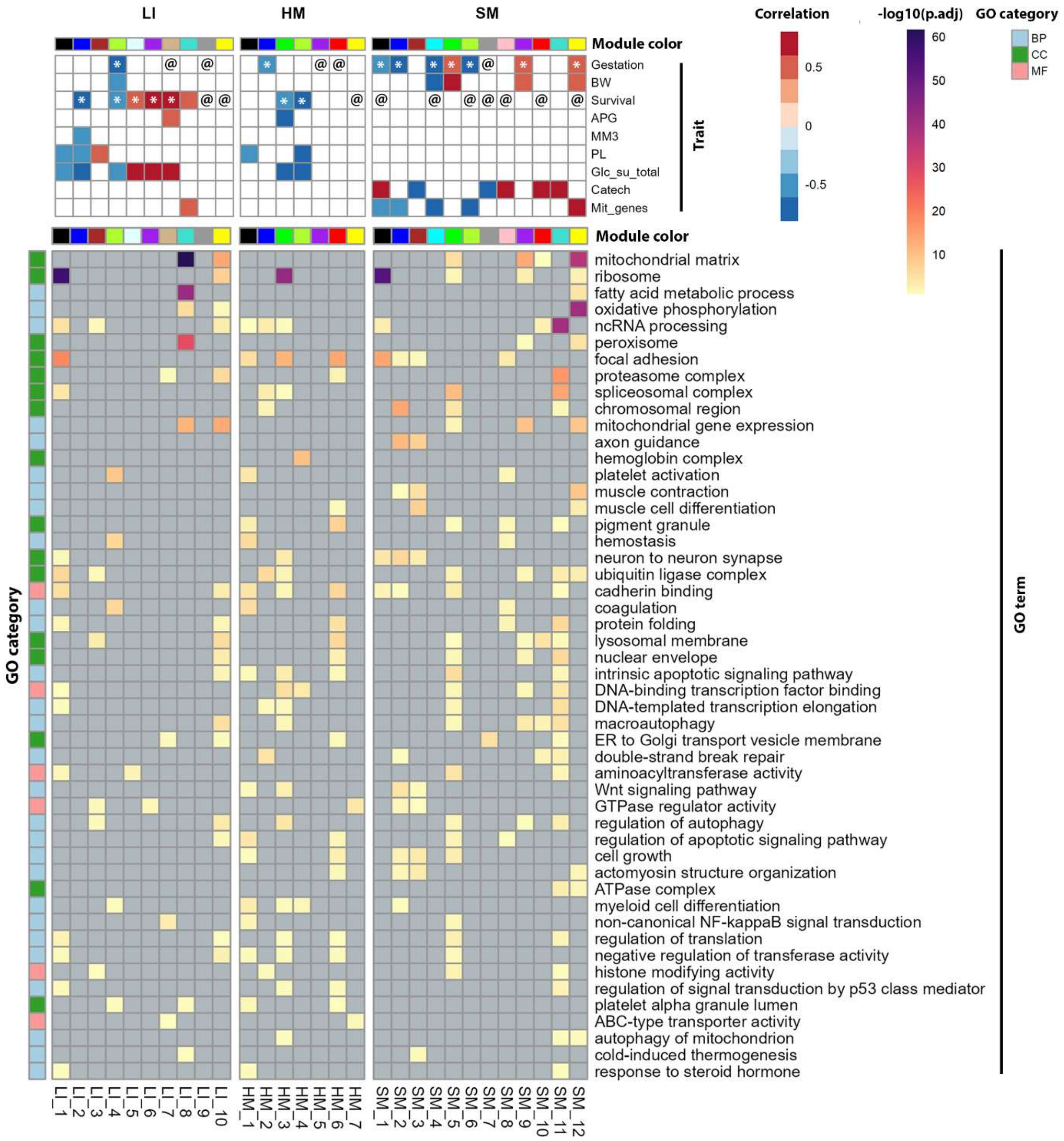

S3 Figure *Selected gene co-expression modules, their correlations with tested traits and functional annotations using ORA and GO database*. See Fig. 8 and Supplementary Table S10 for details.

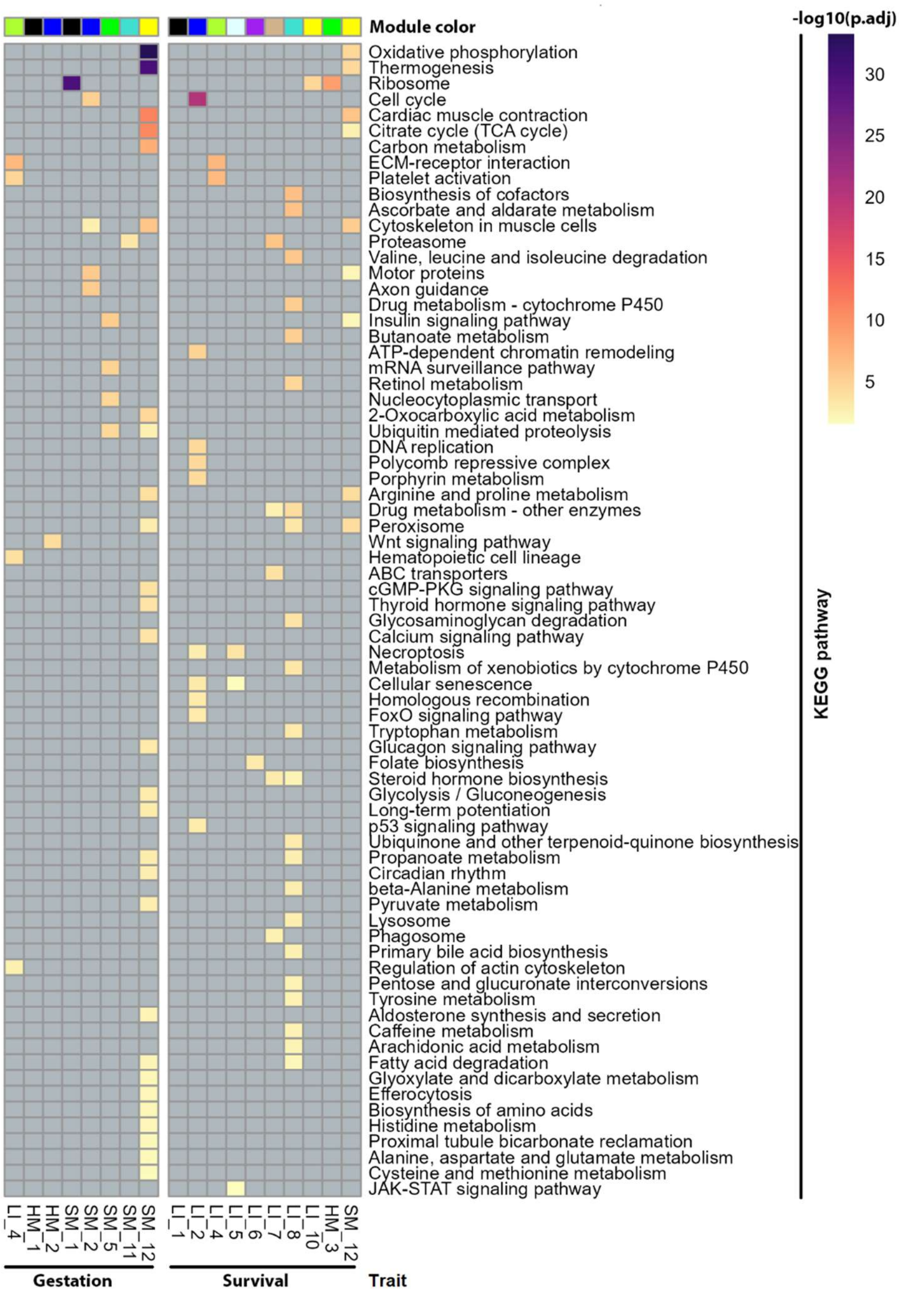

S4 Figure *Functional annotations of genes in Selected modules with verified effect of Gestation or Survival.*

See Fig. 10. Each column represents a distinct gene co-expression module, each assigned a color according to WGCNA convention. Unique module identifiers and the effect of either Gestation or Survival) are indicated at the bottom of each panel. For annotations (lines), see Supplementary Table S11.

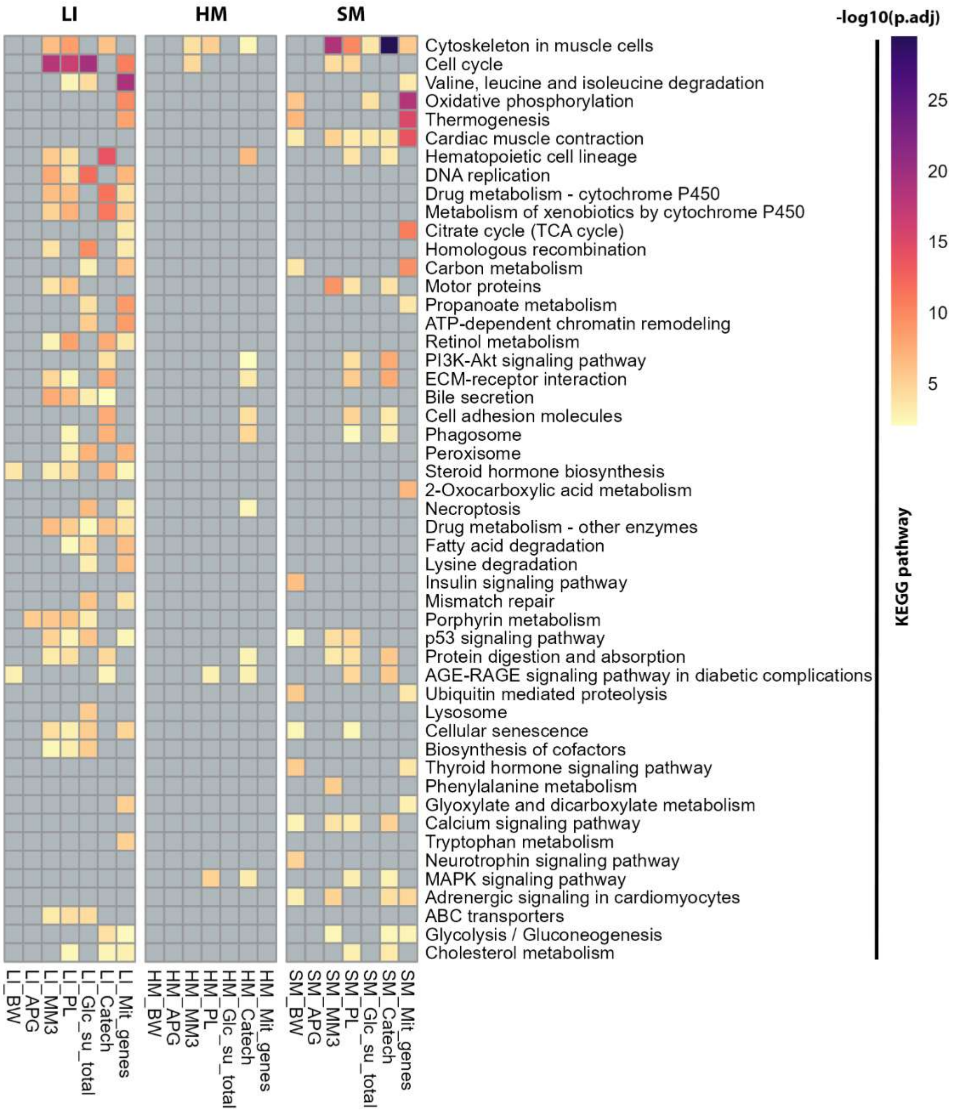

S5 Figure *Functional annotation of DEGs associated birth weight, APG, MM3, PL, Glc_su_total, Catech, and Mit_genes using ORA and KEGG database.* Tissues and traits are shown in columns and annotations in rows. The annotation focused on biochemical pathways using KEGG database. For abbreviations, see Fig. 8. For complete results of the annotations, see Supplementary Tables S12 and S13.
